# Stromal Protein Chloroplast Development and Biogenesis1 Is Essential for Chloroplast Development and Biogenesis in *Arabidopsis thaliana*

**DOI:** 10.3389/fpls.2022.815859

**Published:** 2022-02-10

**Authors:** Weijie Chen, Jingang Huang, Shiwei Chen, Lin Zhang, Jean-David Rochaix, Lianwei Peng, Qiang Xin

**Affiliations:** ^1^Shanghai Key Laboratory of Plant Molecular Sciences, College of Life Sciences, Shanghai Normal University, Shanghai, China; ^2^Departments of Molecular Biology and Plant Biology, University of Geneva, Geneva, Switzerland

**Keywords:** chloroplast, ribosome, mitochondria, CDB1, CDB1L

## Abstract

Although numerous studies have been carried out on chloroplast development and biogenesis, the underlying regulatory mechanisms are still largely elusive. Here, we characterized a chloroplast stromal protein Chloroplast Development and Biogenesis1 (CDB1). The knockout *cdb1* mutant exhibits a seedling-lethal and ivory leaf phenotype. Immunoblot and RNA blot analyses show that accumulation of chloroplast ribosomes is compromised in *cdb1*, resulting in an almost complete loss of plastid-encoded proteins including the core subunits of the plastid-encoded RNA polymerase (PEP) RpoB and RpoC2, and therefore in impaired PEP activity. Orthologs of CDB1 are found in green algae and land plants. Moreover, a protein shows high similarity with CDB1, designated as CDB1-Like (CDB1L), is present in angiosperms. Absence of CDB1L results in impaired embryo development. While CDB1 is specifically located in the chloroplast stroma, CDB1L is localized in both chloroplasts and mitochondria in *Arabidopsis*. Thus, our results demonstrate that CDB1 is indispensable for chloroplast development and biogenesis through its involvement in chloroplast ribosome assembly whereas CDB1L may fulfill a similar function in both mitochondria and chloroplasts.

## Introduction

Chloroplasts are the sites of photosynthesis in eukaryotic cells and arose from a cyanobacterium-like ancestor through endosymbiosis. In higher plants, light triggers chloroplast development from undifferentiated small organelles called proplastids in meristematic cells (Pogson and Albrecht, 2011). During differentiation and biogenesis, chloroplasts transcribe their own genome into mRNAs for protein synthesis. These proteins are essential for the development of functional chloroplasts (Sakamoto et al., 2008). Chloroplast genomes of green plants comprise ~120 genes encoding the components of the gene expression system (RNA polymerase core subunits, ribosomal proteins, tRNAs, and rRNAs), subunits of the photosynthetic machinery [Rubisco, photosystem I and II (PSI and PSII), cytochrome *b_6_f* complex (Cyt *b_6_f*), ATP synthase, and NAD(P)H dehydrogenase-like (NDH) complex] as well as some other proteins involved in various metabolic processes in chloroplasts (Dobrogojski et al., 2020). In addition to the plastid-encoded proteins, chloroplasts also contain ~3,000 nucleus-encoded proteins which are synthesized on cytosolic ribosomes and then imported into chloroplasts (Friso et al., 2004; Heazlewood et al., 2006). Thus, chloroplast development and biogenesis require tight coordination of plastid and nuclear gene expression.

Chloroplast transcription involves the interplay of two types of RNA polymerases: nuclear-encoded phage-type RNA polymerase (NEP) and plastid-encoded bacterial-type RNA polymerase (PEP; Shiina et al., 2005; Börner et al., 2015). In *Arabidopsis*, NEP is encoded by the nuclear genes *RpoTp* and *RpoTmp*, and is essential for transcription of the plastid PEP transcription machinery including *rpoA* and *rpoB-C1-C2* as well as some plastid housekeeping genes (Steiner et al., 2011; Pfalz and Pfannschmidt, 2013). Plastid genes involved in photosynthesis are primarily transcribed by the PEP transcription machinery (Swiatecka-Hagenbruch et al., 2007; Liere et al., 2011). PEP is composed of four catalytic core subunits (α, β, β′, and β″), which are encoded by *rpoA*, *rpoB*, *rpoC1*, and *rpoC2*, respectively. Besides its four core subunits, PEP activity is also regulated by at least 12 PEP-associated proteins (PAPs; Pfalz and Pfannschmidt, 2013). Intriguingly, most mutants of these PEP complex subunits display an albino/ivory leaf phenotype with arrested plastid development and strongly decreased expression of PEP-dependent genes (Pfalz and Pfannschmidt, 2013).

Translation in chloroplasts is performed by prokaryotic-type 70S ribosomes composed of a large 50S and a small 30S subunit. Biochemical analysis showed that, in spinach, the 50S subunit contains 23S, 5S, and 4.5S rRNA as well as 33 ribosomal proteins whereas the 30S subunit consists of 16S rRNA and 25 ribosomal proteins (Manuell et al., 2007; Graf et al., 2016; Perez Boerema et al., 2018). Because of their common origin, chloroplast and bacterial ribosomes exhibit some conserved structural and functional features such as the mRNA decoding (30S small subunit) and the peptide bond synthesis (50S large subunit) functions (Manuell et al., 2007; Sharma et al., 2007). Besides the classical ribosomal proteins which have orthologs in *Escherichia coli*, five plastid-specific ribosomal proteins (PSRPs) with essential functional roles were also found in plant plastid ribosomes (Sharma et al., 2010; Tiller et al., 2012). Although the overall structural organization of chloroplast ribosomes has been characterized, little is known about the molecular mechanisms underlying their assembly.

Ribosome biogenesis is a complicated process that comprises the transcription of a large pre-rRNA precursor, its processing and folding and the assembly of the ribosomal proteins with the mature rRNAs (Shajani et al., 2011; Weis et al., 2015). So far, a dozen of factors appears to be required for the maturation of rRNAs in chloroplasts, such as RNase R homolog 1 (RNR1) which is involved in the maturation of 23S, 16S, and 5S rRNAs (Bollenbach et al., 2005). Loss of endonucleases RNase E in *Arabidopsis* causes defective rRNA processing and subsequent plastid ribosome deficiency (Schein et al., 2008; Walter et al., 2010). The DEAD-box RNA helicases RH22 and RH39 function in the assembly of the 50S ribosomal subunit and 23S rRNA processing, respectively (Nishimura et al., 2010; Chi et al., 2012). A conserved protein with an unknown functional DUF177 domain is specifically required for the accumulation of 23S rRNA (Yang et al., 2016). In addition to the factors required for chloroplast rRNA maturation, there are many factors involved in the biogenesis of chloroplast ribosomes. For example, ObgC is a GTPase that associates with chloroplast 50S ribosomal subunits through 23S rRNA (Bang et al., 2012). Pro-rich protein CGL20 is also required for the assembly of the 50S ribosomal subunit (Reiter et al., 2020).

Here, we have characterized an *Arabidopsis* mutant that displays an ivory leaf phenotype with arrested chloroplast development. We designated this mutant *chloroplast development and biogenesis1* (*cdb1*). The *CDB1* gene encodes a protein with unknown function that is targeted to the chloroplast stroma. Loss of *CDB1* leads to defects in accumulation of plastid-encoded proteins, including chloroplast ribosomes. These data suggest that CDB1 is indispensable for chloroplast development through its involvement in chloroplast ribosome assembly. We also provide evidence that the paralog of CDB1, CDB1-Like (CDB1L), is located both in chloroplasts and mitochondria and essential for embryo development in *Arabidopsis*.

## Materials and Methods

### Plant Material and Growth Conditions

Mutants of *cdb1* (SALK_080811C) and *cdb1l* (GK-844F05) were obtained from the Nottingham *Arabidopsis* Stock Center (NASC). The T-DNA insertions were confirmed by PCR analysis and subsequent sequencing with the primers CDB1-TF and CDB1-TR, CDB1L-TF, and CDB1L-TR (for all primer sequences, see Supplementary Table 3), respectively. Seeds were planted in Murashige and Skoog (MS) culture medium (pH 5.8) with 3% sucrose and 0.7% agar at 4°C in the dark for 48 h. Then the plants were cultured under long-day conditions (16 h-light/8 h-dark) at 23°C with an irradiance of 50 μmol photons m^−2^s^−1^ for 3–4 weeks. For complementation of the mutants, genomic sequences of *CDB1* (*AT4G37920*) and *CDB1L* (*AT1G36320*) plus the upstream promoters were cloned into pCAMBIA1301 to produce transgenic lines. The genomic sequence of *CDB1* was fused with the HA tag and cloned into the pCAMBIA1301 vector to generate *CDB1-HA* transgenic plants. All the above vectors were transferred into *Agrobacterium tumefaciens* strain GV3101 and then introduced into *Arabidopsis* by the floral-dip method (Clough and Bent, 1998).

### Transmission Electron Microscopy

Leaves from 2-week-old plants grown on MS medium with 3% sucrose were fixed with 2.5% glutaraldehyde in phosphate buffer (pH 7.2) for 24 h at 4°C. After washing three times with the same buffer, the fixed samples were dehydrated with a series of ethanol solutions (15, 30, 50, 70, 80, 90, and 100%). Then, the dehydrated samples were infiltrated with a series of epoxy resin in epoxy propane (25, 50, 75, and 100%), and embedded in Epon 812 resin. The samples were cut through an ultramicrotome, and images were taken with a transmission electron microscope (Phillips CM120).

### Subcellular Localization of Green Fluorescent Protein

To study the subcellular location of CDB1 and CDB1L, full cDNA sequences of *CDB1* and *CDB1L* were cloned into the pBI221-GFP vector. The chloroplast localization control RbcS-GFP was constructed as described previously (Zhang et al., 2018). The constructs were transformed into *Arabidopsis* protoplasts by PEG-mediated protoplast transformation. The mitochondrial marker MitoTracker Red CMXRos at a final concentration of 100 nM was incubated with the protoplasts for 15 min in the dark and washed twice before imaging. GFP signals were captured by confocal laser scanning microscopy (LSM 780, Zeiss). The experiment was repeated twice independently with similar results.

### Antibody Production and Antibody Source

Sequences encoding the mature CDB1 and CDB1L proteins were amplified and cloned into the pET-28a expression vector (Merck Millipore) to express the recombinant proteins in the *E. coli* strain BL21 (DE3) in the presence of 0.5 mM IPTG. Recombinant proteins were purified using Ni-NTA agarose (Qiagen) and used to produce rabbit polyclonal antiserum (PhytoAB). Antiserum was employed in dilutions of 1:1,000. For examination of the specificity of CDB1 and CDB1L antibodies, immunoblots were performed using the recombinant CDB1 and CDB1L proteins. No cross reaction between the two proteins was detected when as much as 8 ng of recombinant CDB1 and CDB1L proteins was loaded (Supplementary Figure 3), indicating the specificity of these two antibodies.

Antibodies against HA tag (PhytoAB, PHY5011), D1 (PhytoAB, PHY0057), D2 (PhytoAB, PHY0323), LHCII (made in our lab), PetA (PhytoAB, PHY0321), PetC (PhytoAB, PHY0163), PetD (PhytoAB, PHY0354), PsaA (PhytoAB, PHY0342), PsaD (PhytoAB, PHY0343), CF_1_γ (PhytoAB, PHY0161), CF_1_ε (PhytoAB, PHY0315), RbcL (PhytoAB, PHY0066), phosphoglycerate kinase (PGK1; PhytoAB, PHY0405), ribulose phosphate epimerase (RPE; PhytoAB, PHY0616), RpoB (PhytoAB, PHY1700), RpoC2 (PhytoAB, PHY0382), RPS2 (PhytoAB, PHY0427), RPS4 (PhytoAB, PHY0428), PSRP2 (PhytoAB, PHY0420), RPL1 (PhytoAB, PHY0421), RPL6 (PhytoAB, PHY0411), RPL10 (PhytoAB, PHY0423), RPL11 (PhytoAB, PHY0413), and RPL18 (PhytoAB, PHY0414) were purchased from a commercial supplier and used at a 1:1,000 dilution.

### Protein Isolation and Immunoblot Analysis

Chloroplast stromal proteins and thylakoid membranes were extracted from 4-week-old plants. Intact chloroplasts were isolated using isolation buffer (0.33 M sorbitol and 20 mM HEPES/KOH, pH 7.6) and then osmotically ruptured in 20 mM HEPES/KOH (pH 7.6; Zhang et al., 2018). To separate the thylakoid membranes and stromal proteins, the ruptured chloroplasts were centrifuged at 12,000 × *g* for 10 min at 4°C. Clear supernatant containing stromal proteins was quantified with a Protein Assay Kit (Bio-Rad Laboratories), and the pellet containing thylakoid membrane was solubilized in 20 mM HEPES/KOH (pH 7.6). Both stromal and thylakoid proteins were solubilized in 2 × sample buffer (50 mM Tris–HCl, pH 6.8, 5% SDS, 20% glycerol, 8 M urea, 5% 2-mercaptoethanol, and 1% bromophenol blue).

Mitochondria were isolated as previously described (Sweetlove et al., 2007). Four week-old WT plants were fully homogenized in isolation buffer (0.3 M sucrose, 5 mM tetrasodium pyrophosphate, 10 mM KH_2_PO_4_, pH 7.5, 2 mM EDTA, 1% PVP40, 1% BSA, 5 mM cysteine, and 20 mM ascorbic acid), filtered through Miracloth, and then centrifuged at 5,000 × *g* for 10 min at 4°C. The supernatant was collected and centrifuged at 20,000 × *g* for 10 min at 4°C. The pellet containing crud mitochondria was resuspended in buffer with 0.3 M sucrose, 1 mM EGTA, and 10 mM MOPS/KOH, pH 7.2 and further centrifuged through a Percoll density gradient consisting of 18, 25, and 50% Percoll solution at 40,000 × *g* for 55 min at 4°C. Intact mitochondria at the 25%–50% Percoll interface were collected. Protein concentration was determined using a DC Protein Assay kit (BioRad, 5000116). Mitochondrial proteins were solubilized in 2 × sample buffer.

Protein samples were separated by SDS-PAGE and transferred to nitrocellulose membranes. The membrane was incubated with specific antibodies, and then the signals were detected by the LuminoGraph WSE-6100 (ATTO Technology).

### RNA Sequencing and Differential Gene Expression Analysis

Total RNA was extracted from WT and *cdb1* leaves using Trizol reagent kit (Invitrogen, Carlsbad, CA, United States), and rRNAs were removed by Ribo-Zero™ Magnetic Kit (Epicentre, Madison, WI, United States) to retain mRNAs and other RNAs. Enriched RNAs were fragmented and reverse transcribed into cDNA with random primers. Second-strand cDNA was synthesized, end repaired, and ligated to Illumina sequencing adapters using NEB#7490 kit (NEB E7490L, New England Biolabs). The ligation products of 300–500 bp were selected by agarose gel electrophoresis, PCR amplified, and sequenced using Illumina HiSeq2500 (Gene Denovo Biotechnology Co. Ltd.).

Raw Reads were filtered by fastp (version 0.18.0) to get high quality clean reads (Chen et al., 2018). Clean reads were mapped to the *Arabidopsis* reference genome (TAIR10) using HISAT2.2.4 (Kim et al., 2019) and then sorted according to chromosome and physical position of the reference genome by samtools (Li et al., 2009). Reads were counted by featureCounts in Rsubread (version: 2.2.6) R package and normalized to get Transcripts Per Kilobase of exon model per Million mapped reads (TPM) and trimmed mean of M value (TMM; Liao et al., 2019). Differentially expressed genes (DEGs) were analyzed using DESeq2 (version: 1.28.1) applying a |log_2_(FC)| > 1 and an adjusted *p* < .05 parameters (Love et al., 2014). Gene Ontology (GO) enrichment analysis for DEGs was performed using clusterProfiler package (Wu et al., 2021). A false discovery rate (FDR) of <0.05 was considered for threshold.

The RNA-seq data have been uploaded in the NCBI Sequence Read Archive under accession number PRJNA781386.

### RNA Blot Analysis

Total RNA of WT and *cdb1* was extracted from 4-week-old *Arabidopsis* plants using Trizol reagent (Thermo Fisher Scientific). The rRNAs were detected by ethidium bromide staining. A total of 5 μg RNA of WT and *cdb1* samples were separated by electrophoresis on 1.4% (w/v) agarose-formaldehyde gels and subsequently blotted onto a nylon membrane (GE Healthcare). RNA was fixed on the nylon membrane by UV irradiation (UVP Hybridizer Oven). The membrane was hybridized with the specific probes labeled with digoxigenin, and the signals were visualized by the LuminoGraph WSE-6100 (ATTO Technology). Primers used to generate probes for the chloroplast *rrn* operons are shown in Supplementary Table 3.

### Other Methods

Chlorophyll fluorescence was measured using the MAXI version of the Imaging-PAM M-Series chlorophyll fluorescence system with default settings. Before measurement, the plants were kept in the dark for 20 min. Protein alignment and evolutionary tree were produced using MEGA6 (Tamura et al., 2013).

## Results

### *Arabidopsis cdb1* Mutant Exhibits an Ivory Phenotype

**Figure 1:**
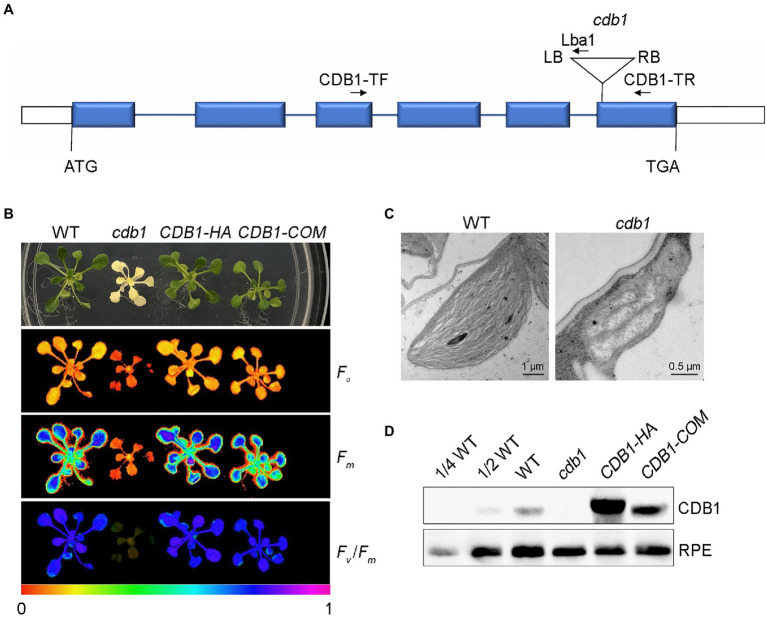
Characterization of the *cdb1*mutant. **(A)** Scheme of the structure of the *Chloroplast Development and Biogenesis1* (*CDB1*) gene with the T-DNA insertion. The six blue boxes indicate exons. 5′ and 3′ UTRs are shown as white boxes and introns as black lines. The T-DNA insertion is indicated by a triangle. The forward (CDB1-TF) and reverse primers (CDB1-TR and Lba1) used for PCR analysis are indicated with arrows. **(B)** Chlorophyll fluorescence images of 4-week-old WT (wild-type), *cdb1* and complemented plants *CDB1-HA* and *CDB1-COM*. The minimal fluorescence (*F*_o_), the maximal fluorescence (*F*_m_) and *F*_v_/*F*_m_ of WT, *cdb1* and two complemented plants were measured by Imaging-PAM. Fluorescence was visualized using a pseudocolor index from red (0) to purple (1) as indicated at the bottom. **(C)** Transmission electron micrographs of chloroplasts in leaves from 2-week-old WT and *cdb1* mutant. Scale bars are indicated. **(D)** Protein accumulation of CDB1 in total protein extracts from 4-week-old leaves of WT, *cdb1* and complemented plants *CDB1-HA* and *CDB1-COM*. Immunoblotting was performed with a CDB1-specific antibody. An antibody specific for ribulose phosphate epimerase (RPE) was used as loading control.

In recent years, many mutants with albino or ivory phenotype were reported. Analysis of these mutants by transmission electron microscopy revealed defects in plastid development and biogenesis as for *pdm4* and several *pTAC* mutants (Pfalz et al., 2006; Wang et al., 2020). To further investigate the underlying mechanisms of chloroplast development and biogenesis, we characterized a T-DNA insertion mutant (SALK_080811C) with an ivory phenotype (Figures 1A,B). PCR product sequencing showed that the T-DNA was inserted in the sixth exon of *AT4G37920* (Figure 1A). This mutant could grow on MS medium supplemented with 3% sucrose but could not survive photoautotrophically when transplanted in soil. The leaves of this mutant display an ivory phenotype (Figure 1B). By transmission electron microscopy observation, we found that, unlike the chloroplasts in WT with well-organized thylakoid membranes, the plastids of this mutant did not exhibit organized membrane structures (Figure 1C). These results indicate that chloroplast development and biogenesis is arrested. Accordingly, we named this mutant *cdb1*.

Chlorophyll fluorescence of WT and *cdb1* plants grown on MS medium was analyzed. In WT, the minimal fluorescence (*F*_o_) and the maximal fluorescence (*F*_m_) were 0.17 ± 0.01 and 0.75 ± 0.01, respectively. The maximum quantum efficiency of PSII (*F*_v_/*F*_m_) was 0.77 ± 0.01. Consistent with the ivory phenotype of *cdb1*, almost no chlorophyll fluorescence was detected in the *cdb1* mutant as *F*_o_ and *F*_m_ were 0.069 ± 0.020 and 0.086 ± 0.020, respectively (Figure 1B). The value of *F*_v_/*F*_m_ is close to 0, indicating no PSII activity in *cdb1*.

To confirm that the ivory leaf phenotype was due to the disruption of *CDB1* in *cdb1*, we complemented the mutants with the genomic gene sequence including its native promoter (*CDB1-COM*) and the full-length coding region of *CDB1* fused to the HA tag at its C terminus (*CDB1-HA*). The *CDB1-COM* and *CDB1-HA* complemented plants displayed a phenotype similar to WT and the ivory leaf and fluorescence emission defects were all rescued (Figure 1B). A specific antibody raised against CDB1 was used to detect the CDB1 protein by immunoblotting using a total protein extract from 4-week-old leaves of WT, *CDB1-HA*, and *CDB1-COM* plants (Figure 1D). The *CDB1* gene encodes a 48.73 kDa protein with a predicted chloroplast transit peptide (cTP). A signal corresponding to a molecular mass of about 42 kDa was detected consistent with the predicted molecular mass of CDB1 without cTP (Figure 1D). CDB1-HA fusion protein was detected in the *CDB1-HA* complemented plants with a slightly larger molecular mass than the native CDB1 protein (Figure 1D). CDB1 was absent in the *cdb1* mutant due to the T-DNA insertion in *AT4G37920*. These results indicate that *cdb1* is a null mutation and *CDB1* is responsible for chlorophyll accumulation and chloroplast development.

### Chloroplast Protein Accumulation in *cdb1*

**Figure 2:**
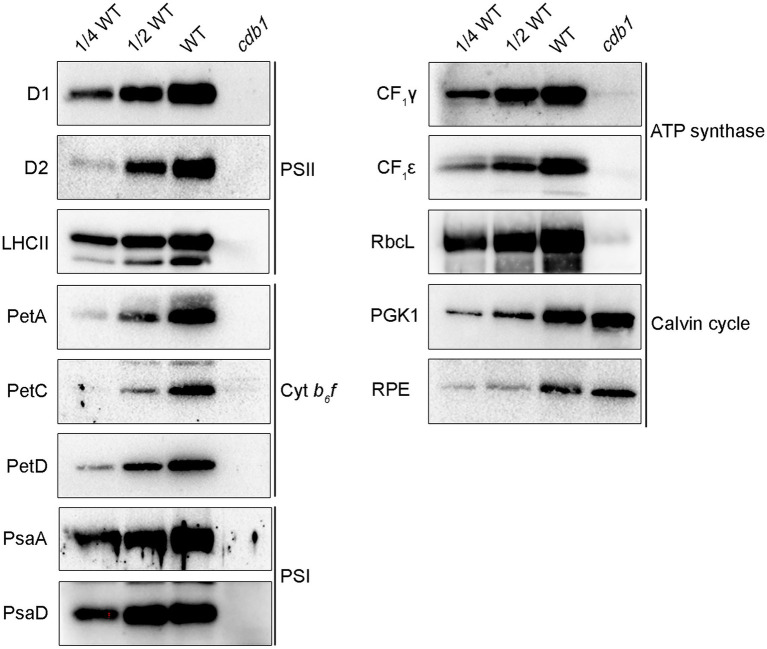
Comparative analysis of subunits of photosynthetic complexes from WT and *cdb1*. Total protein isolated from 3-week-old WT and *cdb1* plants was analyzed by immunoblotting with antibodies against representative subunits of the multiprotein complexes of the thylakoid membrane: D1, D2, and LHCII from photosystem II (PSII), PetA, PetC, and PetD from Cyt *b_6_f*, PsaA and PsaD from PSI, CF_1_γ, and CF_1_ε from ATP synthase. Antibodies against RbcL, phosphoglycerate kinase (PGK1), and RPE were used as representative components of the Calvin cycle.

Since the *cdb1* mutant showed an ivory phenotype, it was necessary to investigate chloroplast protein accumulation *in vivo*. Total protein from 3-week-old leaves of WT and *cdb1* was isolated and tested by immunoblot analysis using antibodies against subunits of the major photosynthetic complexes. Accumulation of thylakoid membrane proteins, including D1, D2, and LHCII of the PSII complex, PetA (Cyt *f*) and PetD of Cyt *b_6_f*, and PsaA and PsaD of PSI were all undetectable in the *cdb1* mutant and only trace amounts of CF_1_γ and CF_1_ε of ATP synthase and PetC of Cyt *b_6_f* were detected (Figure 2). Abundance of three enzymes involved in the photosynthetic carbon reduction Calvin cycle was also analyzed (Figure 2). While a trace amount of plastid-encoded RuBisCO large subunit (RbcL) was detected in the *cdb1* mutant, the levels of the nuclear-encoded PGK1 and RPE were comparable in the mutant to those of WT (Figure 2).

These results indicate that all plastid-encoded proteins analyzed do not accumulate or are only present in tiny amounts in *cdb1* as shown for D1, D2, PetA, PsaA, CF_1_ε, and RbcL. Nucleus-encoded chloroplast proteins whose stable accumulation is independent of the presence of other plastid-encoded proteins were present in normal amounts in the *cdb1* mutant as seen for PGK1 and RPE. These results agree with earlier studies showing that the stable accumulation of proteins belonging to a photosynthetic complex depends on the presence of all core subunits and especially on those that are plastid-encoded (Naver et al., 2001; Peng et al., 2006). Therefore, a possible explanation for the immunoblot results of *cdb1* is that synthesis of all plastid-encoded proteins is blocked in *cdb1* resulting not only in the absence of these proteins but also of their nucleus-encoded partner proteins of the same photosynthetic complex. In contrast, other nucleus-encoded chloroplast proteins such as PGK1 and RPE are unaffected in *cdb1* (Figure 2).

### RNA-Seq Analysis of the *cdb1* Mutant

**Figure 3:**
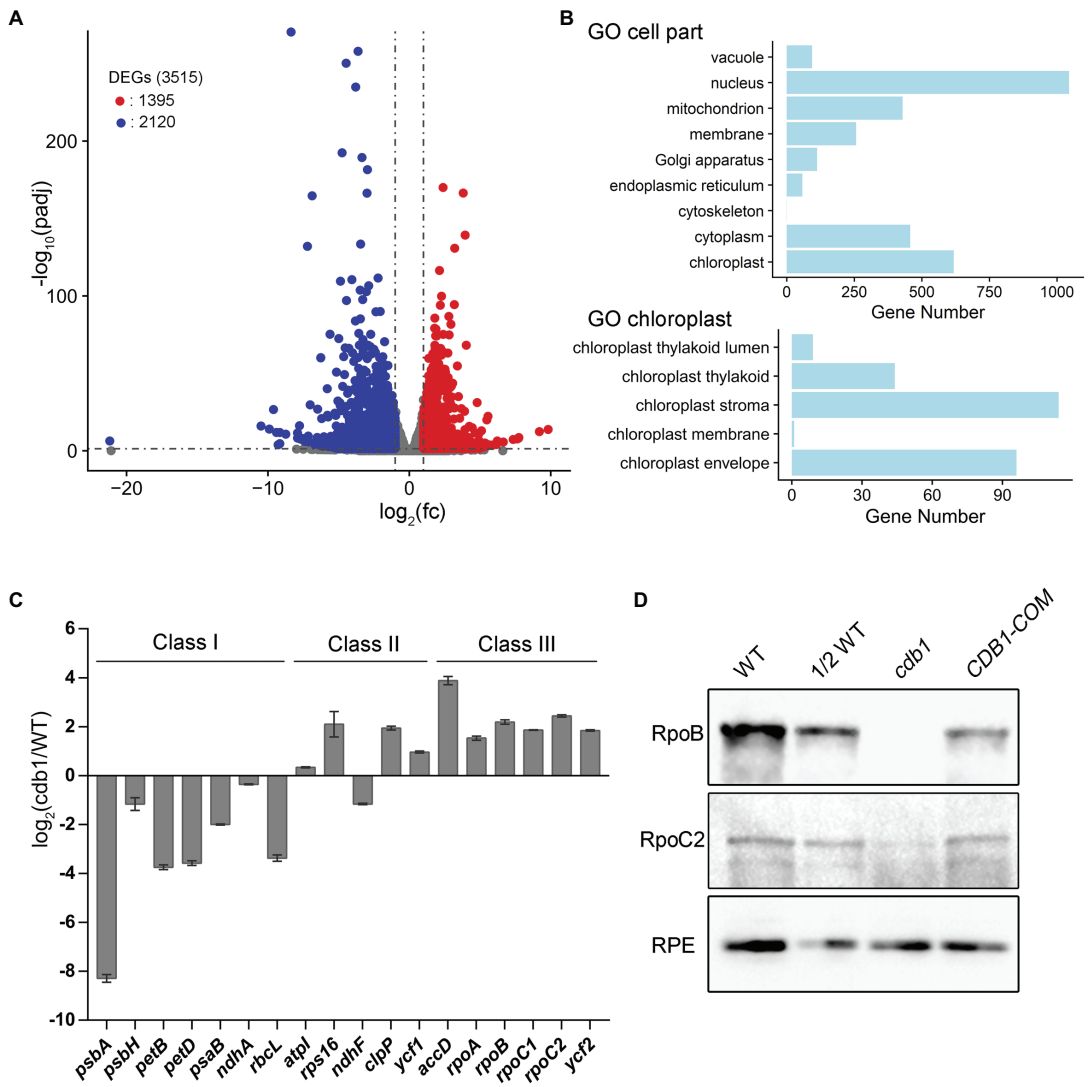
Transcriptome analysis of WT and *cdb1* plants. **(A)** Volcano plot showing the differentially expressed genes (DEGs) in WT and *cdb1* [*p adj* < 0.05, fold change (fc) > 2]. Total RNA was extracted from 4-week-old leaves of *cdb1* and WT. The constructed cDNA library was sequenced using lllumina HiSeq 2,500. DEGs were identified by DESeq2 according to the parameter of fold change >2 and adjusted *p* value below 0.05. Volcano plot was generated by R package ggplot2. Down- and upregulated genes are indicated by blue and red dots, respectively. Three biological replicates of WT and *cdb1* were used for RNA-seq experiment. **(B)** Diagram showing gene numbers relative to “cell part” and “chloroplast” in Gene Ontology (GO) enrichment of DEGs in *cdb1* vs. WT. GO enrichment of DEGs was performed using clusterProfiler. **(C)** Plastid gene transcript levels. Differential expression of plastid genes in *cdb1* vs. WT is represented according to the RNA-seq data. The representative plastid genes include Class I genes (*psbA*, *psbH*, *petB*, *petD*, *psaB*, *ndhA*, and *rbcL*), Class II genes (*atpI*, *rps16*, *ndhF*, *clpP*, and *ycf1*), and Class III genes (*accD*, *rpoA*, *rpoB*, *rpoC1*, *rpoC2*, and *ycf2*). Log_2_[fold change(*cdb1*/WT)] ± *SD* values are from three biological replicates. **(D)** Immunoblot analysis of RpoB and RpoC2. Total protein isolated from 3-week-old WT, *cdb1* and *CDB1-COM* were hybridized with antibodies against RpoB and RpoC2. RPE was used as a loading control.

To determine the function of CDB1 in chloroplast development, we carried out an RNA-seq analysis on 4-week-old leaves of *cdb1* and WT. Transcriptional profiles of nucleus- and chloroplast-encoded genes were compared using RNA-seq. A total of 3,515 genes were differentially expressed more than 2-fold in *cdb1* compared to WT (Figure 3A). They include 1,395 upregulated and 2,120 downregulated genes (Figure 3A). Gene Ontology (GO) analysis revealed that a large proportion of the DEGs are related to the nucleus and chloroplast (Figure 3B). Among 618 DEGs related to the chloroplast (GO:0009507), 272 nuclear and 30 chloroplast DEGs are upregulated whereas 296 nuclear and 18 chloroplast DEGs are downregulated (Supplementary Figure 1).

The plastid genes can be divided into three classes according to which plastid RNA polymerase transcribes them. Class I and III are transcribed by the PEP and NEP, respectively. Transcription of class II depends on both PEP and NEP. RNA-seq results showed that expression of class I genes (e.g., *psbA*, *psbH*, *petB*, *petD*, *ndhA*, and *rbcL*) was significantly reduced whereas expression of Class III genes (e.g., *accD*, *rpoA*, *rpoB*, *rpoC1*, *rpoC2*, and *ycf2*) and most of Class II genes (e.g., *atpI*, *rps16*, *clpP*, and *ycf1*) was greatly upregulated (Figure 3C; Supplementary Table 1). The upregulated NEP-dependent Class III genes and the differentially expressed nuclear genes of chloroplast proteins may possibly participate in a compensating response to the decreased expression of Class I genes. These results suggest that transcription of the PEP-dependent genes is impaired in the absence of CDB1. Immunoblot analysis showed that the core subunits of the PEP complex RpoB is undetectable and RpoC2 is only present in a tiny amount in *cdb1* (Figure 3D), indicating that absence of the functional PEP complex is responsible for the impaired transcription of the PEP-dependent genes in *cdb1*.

### Decreased Accumulation of Chloroplast Ribosomes in *cdb1*

**Figure 4:**
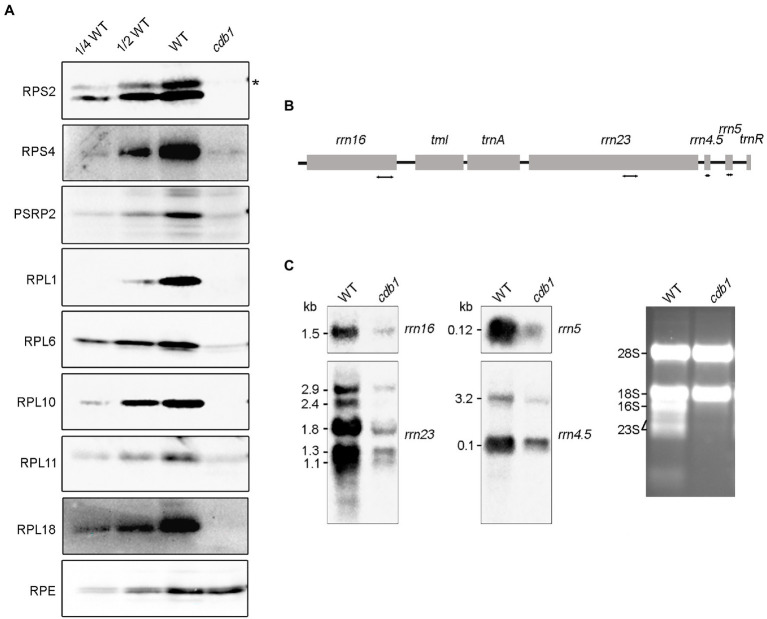
Accumulation of chloroplast ribosomes in WT and *cdb1* plants. **(A)** Immunoblot analysis of representative 50S and 30S ribosomal subunit proteins. Total protein isolated from 3-week-old WT and *cdb1* plants was analyzed by immunoblotting with antibodies against RPS2, RPS4, PSRP2, RPL1, RPL6, RPL10, RPL11, and RPL18. RPE was used as a loading control. Black asterisk indicates the authentic protein. **(B)** Scheme of the chloroplast *rrn* operon with the four probes used for the RNA blots. The four probes for *rrn16*, *rrn23*, *rrn4.5*, and *rrn5* are marked by double-arrow lines under the chloroplast *rrn* operon. **(C)** Accumulation of rRNA in WT and *cdb1*. Total RNA from leaves of 3-week-old WT and *cdb1* was subjected to RNA blot analysis with specific probes against 16S, 23S, 4.5S, and 5S. Sizes of the distinct forms of the rRNA species are indicated on the left; rRNAs of WT and *cdb1* stained with ethidium bromide were used as loading control.

Although drastic reductions of RpoB and RpoC2 were observed in *cdb1*, the levels of their mRNAs are higher in *cdb1* than in WT (Figures 3C,D). This observation implies that translation of *rpoB* and *rpoC2* by ribosomes or assembly of the PEP complex is impaired in *cdb1*. To address the possible role of *CDB1* in chloroplast ribosome accumulation, we examined the levels of chloroplast ribosomal proteins and rRNA in *cdb1* and WT. Immunoblot analysis showed that the amount of 30S ribosomal protein RPS2, RPS4, and PSRP2 and 50S ribosomal subunits RPL1, RPL6, RPL10, RPL11, and RPL18 are barely detectable or greatly reduced in *cdb1* (Figure 4A). Chloroplast rRNAs are transcribed from a single transcription unit that includes the 16S, 23S, 4.5S, and 5S *rrn* genes (Figure 4B). The RNA precursors are then processed to produce mature 16S, 23S, 4.5S, and 5S rRNA (Figure 4B). RNA blotting revealed that the amounts of 1.5-kb 16S, 1.1 and 1.3-kb 23S, 0.1-kb 4.5S, and 0.12-kb 5S mature rRNAs are all drastically reduced (Figure 4C). These results indicate that accumulation of chloroplast ribosomes is impaired in *cdb1*, and explains why accumulation of the chloroplast-encoded proteins is reduced in *cdb1* (Figure 2). Taken together, we propose that CDB1 is essential for the assembly of chloroplast ribosomes.

### Molecular Characterization of the CDB1 Protein

**Figure 5:**
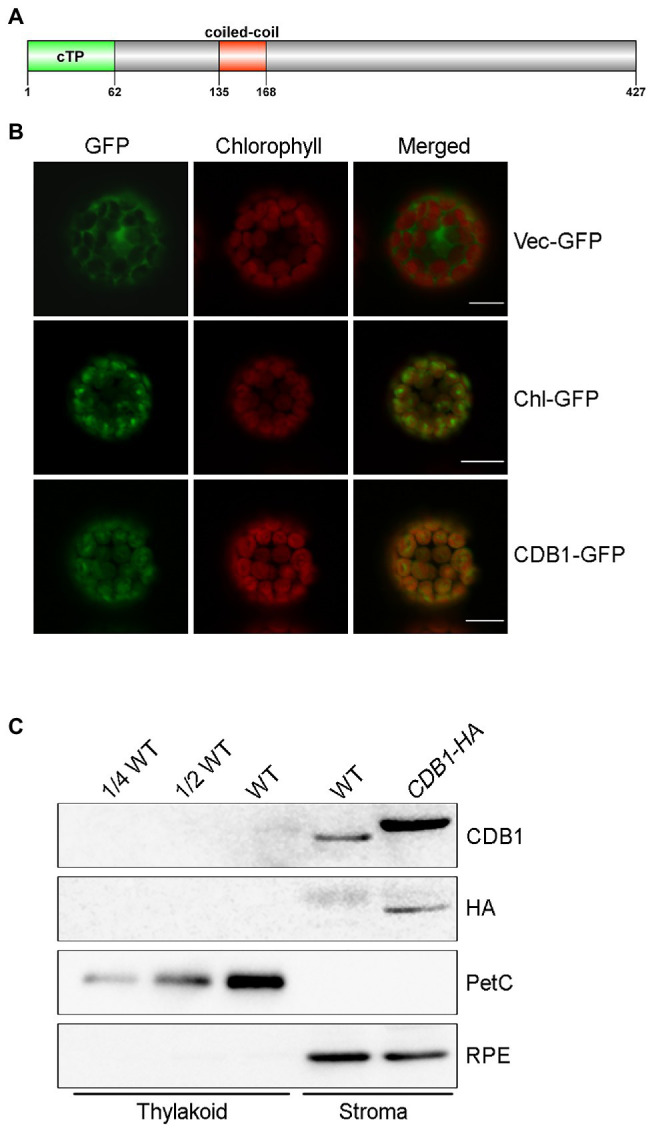
Subcellular localization of CDB1. **(A)** Protein structure of CDB1 protein. The CDB1 protein contains 427 amino acids. The light green part indicates the cTP region; the red box indicates the predicated coiled coil domain. **(B)** Subcellular localization of CDB1 fused with GFP. Images of GFP and chlorophyll fluorescence of protoplasts were captured by confocal laser scanning microscopy (LSM) and merged. The small subunit of ribulose-1, 5-bisphosphate carboxylase fused to GFP (Chl-GFP) was used as chloroplast-targeted control. Bars = 10 μm. **(C)** Immunoblot analysis of CDB1 in chloroplasts. Intact chloroplasts from WT and *CDB1-HA* complemented plants were separated into stroma and thylakoid membrane fractions and reacted with antibodies against CDB1 and HA. PetC and RPE were used as controls for the fractionation of thylakoids and stroma, respectively.

The *CDB1* gene consists of six exons separated by five introns. It encodes a 427-amino acid protein with an unknown function. SMART search-based analysis^1^ and the annotation of The Plant Proteome Database (PPDB)^2^ suggest that CDB1 contains a putative cTP at its N-terminus (1–62 aa) and a predicated coiled-coil domain (135–168 aa; Figure 5A). No other assigned functional motif was found in CDB1.

To determine the subcellular localization of CDB1, a construct containing 35S:CDB1-GFP was transformed into *Arabidopsis* protoplasts. Strong GFP signals were exclusively detected in chloroplasts with CDB1-GFP and they closely merged with chlorophyll autofluorescence (Figure 5B). Besides, the fluorescent signals of CDB1-GFP were similar to those from RbcS-GFP, the small subunit of ribulose-1, 5-bisphosphate carboxylase (RbcS) fused to GFP (Figure 5B). These results indicate that CDB1 is a chloroplast protein, consistent with the presence of cTP at its N-terminus (Figure 5A). To investigate its precise localization, immunoblots were performed using chloroplast stroma and thylakoids isolated from WT and *CDB1-HA* complemented plants. The results show that CDB1 and HA-tagged CDB1 are localized in the chloroplast stroma (Figure 5C).

### Evolution and Structural Analyses of CDB1 in Photosynthetic Viridiplantae

**Figure 6:**
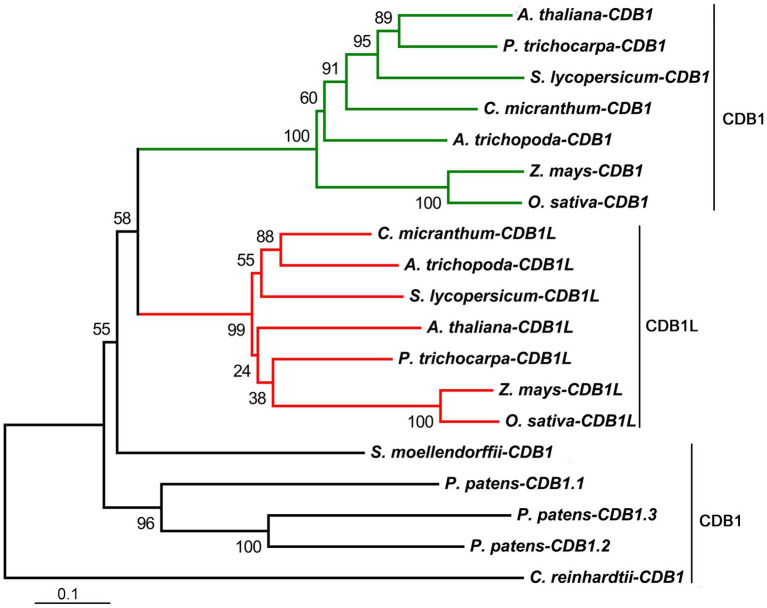
Phylogenetic tree of CDB1 homologs. Nineteen proteins of the CDB1 family were selected to infer the evolutionary history using the Neighbor-Joining method, with bootstrap values (%) from 1,000 replicates. Numbers at branches are percentage of replicate trees in the bootstrap test. The scale bar indicates the units of the number of amino acid substitutions per site. All the analyses were conducted in MEGA6. Alignments of all 19 sequences based on identity/similarity and structural properties are shown in Supplementary Figure 2.

**Figure 7:**
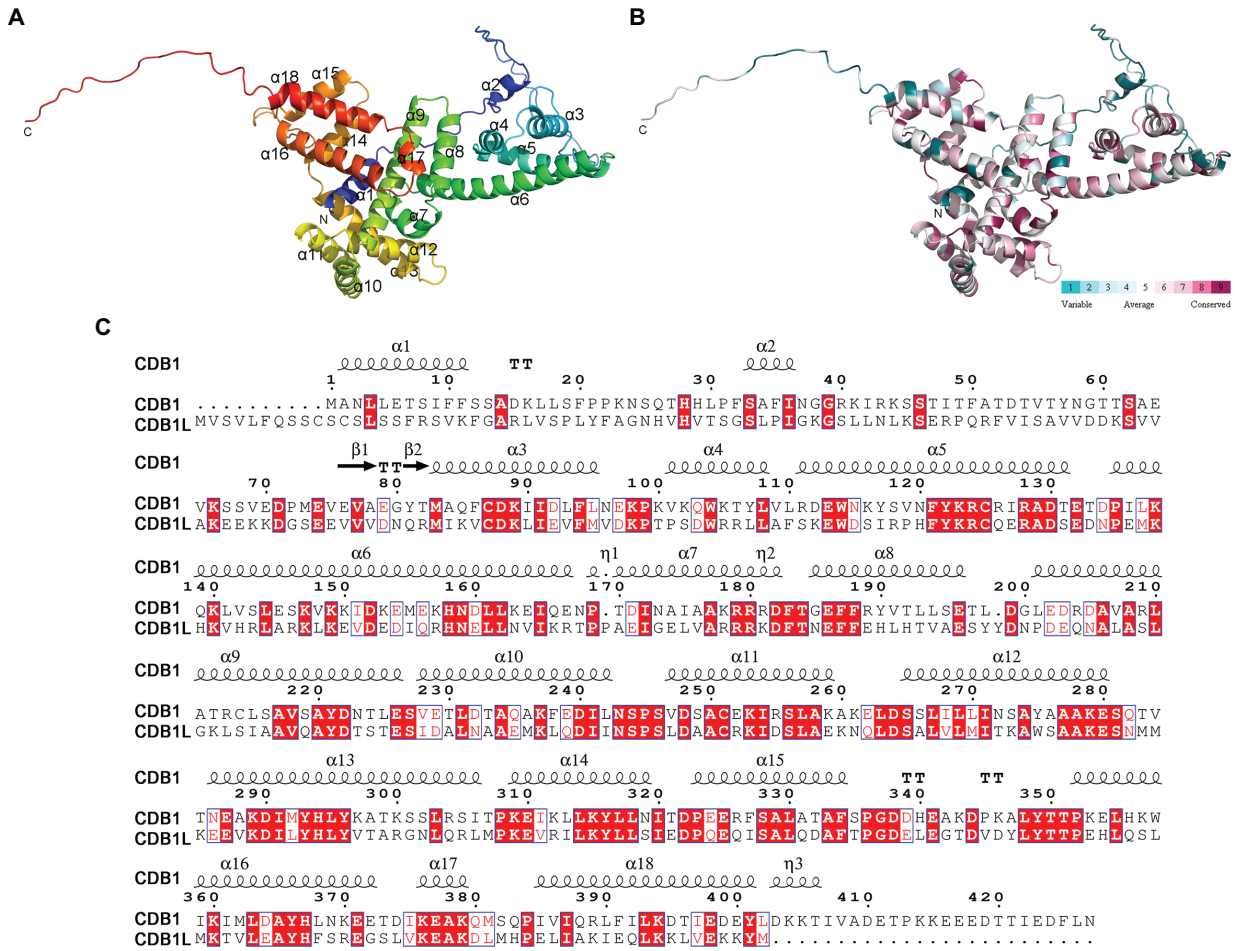
Overall structure of CDB1. **(A)** Crystal structure of CDB1 predicted by AlphaFold2. The α-helices and N, C-terminus are indicated. **(B)** Surface conservation analysis of CDB1 using ConSurf web server. The cartoon structure of CDB1 was colored according to the conservation score from the 19 CDB1 family homologs. **(C)** Sequence alignment of CDB1 and CDB1L from *Arabidopsis*. Secondary structure elements above the alignment were generated using ESPript (https://espript.ibcp.fr). Numbers indicate the original amino acid positions in CDB1. Highly conserved residues are represented by white letters with a red background.

To explore the phylogenic evolution of CDB1, its homologs were searched using BLAST-P against the NCBI database.^3^ Putative orthologs and paralogs were found in most photosynthetic eukaryotes (land plants and green algae) with a high conservation across the entire protein sequence except for the cTP region (Supplementary Figure 2). No homolog of CDB1 was found in cyanobacteria. A neighbor-joining phylogenetic tree for CDB1 homologs was constructed, containing 19 genes from 10 sequenced species representing green algae and land plants (Figure 6). Both green alga *Chlamydomonas reinhardtii* and the lycophyte *Selaginella moellendorffii* have one homolog of *CDB1*. A total of three homologous sequences were found in the moss *Physcomitrella patens*. No homologs were found in gymnosperms probably due to the incomplete genome information. Interestingly, besides CDB1, a second CDB1-related protein, designated as CDB1L, was found from both monocot and dicot plants. The mature *Arabidopsis* CDB1 and CDB1L proteins share 41.5% amino acid sequence identity (Figure 7). These imply that CDB1 proteins originated from green alga and were resolved into two clades in angiosperms during evolution.

The elucidation of the function of CDB1 remains challenging because no homolog or similar domain was characterized in previous reports. To gain insights into this question, the three-dimensional structure of *Arabidopsis* CDB1 was predicted by AlphaFold2^4^ (Jumper et al., 2021; Figure 7A). The predicted structure of mature CDB1 contains two very short β-sheets (β1 and β2) and a total of 17 helical structures, including 14 α-helices (α3–17) and three 3_10_-helices (Figures 7A,C). Based on the sequence identity to CDB1 homologs (Supplementary Figure 2), the evolutionary conservation scores were mapped onto the *Arabidopsis* CDB1 structure using the ConSurf server. The results show that the conserved residues in the CDB1 protein family are clustered in the secondary structure elements of mature *Arabidopsis* CDB1 (Figure 7B; Supplementary Figure 2). This suggests that the members of the CDB1 protein family might have similar biological functions.

### Functional Analysis of CDB1L in *Arabidopsis*

**Figure 8:**
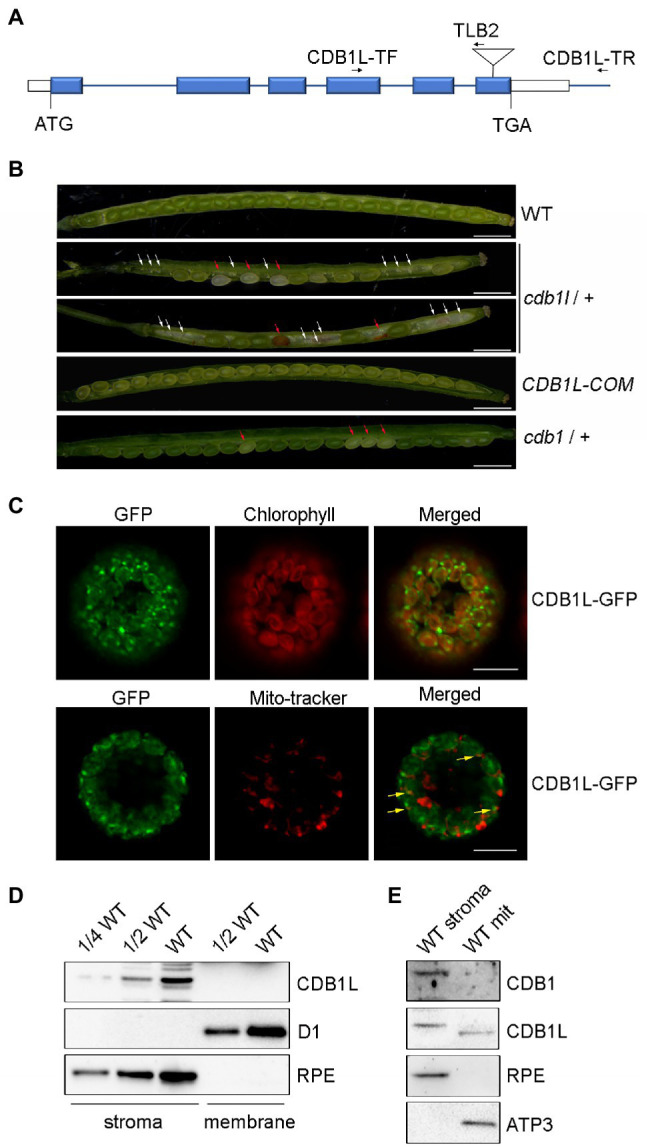
Characterization of the CDB1L in *Arabidopsis*. **(A)** Scheme of the structure of the *CDB1L* gene and its T-DNA insertion. The six blue boxes indicate exons. 5′ and 3′ UTRs are shown as white boxes. The T-DNA insertion is indicated by a triangle. Forward (CDB1L-TF) and reverse primers (CDB1L-TR and TLB2) used for the PCR analysis are indicated with arrows. **(B)** Siliques of WT, *cdb1l* heterozygotes, *CDB1L-COM*, and *cdb1* heterozygotes after flowering. Seeds of early developing siliques of WT and *CDB1L-COM* were all green. Pale and green seeds were observed in early developing siliques of *cdb1l* heterozygous plants (top image of *cdb1l/+*) and of *cdb1* heterozygous plants (*cdb1/+*). The pale seeds became withered at a later stage of seed development in *cdb1l/+* (bottom image of *cdb1l/+*). White arrows highlight the aborted embryos in *cdb1l/+*. The pale and withered seeds in developing *cdb1l/+* and *cdb1/+* siliques are indicated with red arrows. Bars = 1 mm. **(C)** Subcellular localization of CDB1L fused with GFP. CDB1L-GFP, CDB1L-GFP fusion; Mito-tracker, Mito-tracker Red. The co-localization signals of CDB1L-GFP and Mito-tracker are indicated with yellow arrows. Bars = 10 μm. **(D)** Immunoblot analysis of CDB1L in chloroplasts. Intact chloroplasts from WT were separated into stroma and thylakoid membrane fractions and analyzed by immunoblotting with antibodies against CDB1L. D1 and RPE were used as controls for the fractionation of thylakoid and stroma, respectively. **(E)** Immunoblot analysis of CDB1L in mitochondria and chloroplast stroma. Stromal chloroplast and mitochondrial proteins from WT were separated and reacted with antibodies against CDB1 and CDB1L. RPE and ATP3 were used as controls for the fractionation of stroma and mitochondria, respectively.

To investigate the functions of the *CDB1* paralog which was designated as *CDB1L* in *Arabidopsis*, the *cdb1l* (GK-844F05) mutant was obtained. Sequencing of PCR products showed that the T-DNA was inserted into the sixth exon of the *CDB1L* (Figure 8A). Because we were unable to obtain *cdb1l* homozygous mutants, we determined the segregation ratio of heterozygous to WT phenotype in the progeny of *cdb1l* heterozygous plants and found that it was close to 2:1 (Supplementary Table 2). Thus, it is likely that homozygous lethality occurs during embryo development of *cdb1l* homozygous seeds. To test this possibility, we examined the seeds in developing siliques from the *cdb1l* heterozygous plants. A deficiency in endosperm or embryo development of some seeds was detected in these plants (Figure 8B). Some of the affected seeds gave rise to intact seeds but they were pale and became withered at a later stage of development (Figure 8B). It is interesting that some seeds of the *cdb1* heterozygous plants also exhibit a pale color (Figure 8B). These seeds are likely to be the *cdb1* homozygous seeds defective in chloroplast biogenesis.

To confirm that the seed abortion phenotype was due to the disruption of *CDB1L*, we complemented the *cdb1l* heterozygous mutant with the full-length genomic *CDB1L* sequence driven by its authentic promoter. As shown in Figure 8B, the complemented plants (*CDB1L-COM*) produced well-developed seeds similar to WT, confirming that the aborted seeds in the *cdb1l* heterozygous mutants resulted from the disruption of *CDB1L*.

To determine the subcellular localization of CDB1L, a construct containing its coding region fused with GFP at the C-terminus was transformed into *Arabidopsis*. The CDB1L-GFP signal co-localized with chlorophyll (Figure 8C), suggesting that CDB1L is localized in chloroplasts. However, some of the CDB1L-GFP signals were also found outside chloroplasts and co-localized with mito-tracker red, indicating that CDB1L is also localized in mitochondria (Figure 8C). To further confirm the location of CDB1L, a specific antibody against CDB1L was raised to detect CDB1L accumulation in chloroplasts and mitochondria (Figures 8D,E; Supplementary Figure 3). The stroma and thylakoid fractions of chloroplasts isolated from WT leaves were used for immunoblot analysis. The results showed that CDB1L is localized in the chloroplast stroma (Figure 8D). These results are consistent with the localization of CDB1L-GFP (Figure 8C) and the detection of CDB1L in chloroplasts by mass spectrometry (The Plant Proteome Database).^5^

Analysis of proteins from purified mitochondria showed that CDB1L is also present in mitochondria. However, the molecular mass of mitochondrial-localized CDB1L is slightly less than that of chloroplast-localized CDB1L (Figure 8E). This might be due to the different length of the transit peptide in CDB1L, which is removed after entering into chloroplasts and mitochondria. No CDB1 signal was found in the mitochondrial samples (Figure 8E), indicating that CDB1 is not a mitochondrial protein but specifically localized in chloroplasts (Figure 5). In summary, these results indicate that the CDB1L protein has a dual localization in chloroplasts and mitochondria. Loss of CDB1L leads to a deficiency of seed development in *Arabidopsis*.

## Discussion

Chloroplast development is a programmed and complicated process regulated by numerous nuclear and chloroplast genes (Pogson and Albrecht, 2011). Previous studies have identified many nuclear factors that are involved in the regulatory mechanisms of chloroplast development. Here, we report the existence of a novel chloroplast stromal protein CDB1 that is essential for chloroplast development and biogenesis. Absence of CDB1 leads to an ivory seedling phenotype of *Arabidopsis* and the seedlings cannot survive autotrophically in the soil (Figure 1). Consistent with its ivory and chlorophyll fluorescence phenotype, the *cdb1* mutant is defective in the accumulation of thylakoid protein complexes and RuBisCO complex in the chloroplast stroma (Figure 2). These results suggest that CDB1 is essential for chloroplast development and plant growth.

RNA-seq analysis revealed that plastid gene expression was dramatically altered in *cdb1* leaves (Supplementary Figure 1). Transcript levels of PEP-dependent genes were all decreased (Class I genes, including *psbA*, *psbH*, *petB*, *petD*, *psaB*, and *rbcL*); on the contrary, those of NEP-dependent genes were unchanged or even increased (Class III genes, including *rpoA*, *rpoB*, *rpoC1*, *rpoC2*, *clpP*, *ycf1*, and *ycf2*; Figure 3C). These results indicate that *cdb1* mutant is severely impaired in PEP activity. A similar molecular phenotype has been observed in mutants lacking PEP components or regulatory factors, such as *ptac2*/*ptac6*/*ptac12*/*ptac14* (Pfalz et al., 2006; Gao et al., 2012), *sig6* (Loschelder et al., 2006; Chi et al., 2010); PPR genes, such as *pdm2*, *pdm3*, and *pdm4* (Du et al., 2017; Zhang et al., 2017; Wang et al., 2020); and some other factors, such as *ys1* (Zhou et al., 2009), *clb19* (Chateigner-Boutin et al., 2008), and *dg1* (Chi et al., 2008). In contrast to these mutants, however, the *cdb1* mutant accumulates no RpoB and only a trace amount of RpoC2 (Figure 3D), indicating the impaired formation of the PEP complex. Because the expression levels of the genes encoding the core PEP subunits (*rpoA*, *rpoB*, *rpoC1*, and *rpoC2*) are highly increased in *cdb1* (Figure 3C; Supplementary Figure 1), it is likely that the translation of the PEP subunits or assembly of the PEP complex is impaired in the chloroplasts of *cdb1*.

Further immunoblot and RNA analysis showed that the accumulation of proteins of the 30S and 50S ribosomal subunits as well as ribosomal rRNAs (16S, 23S, 4.5S, and 5S) is dramatically decreased in *cdb1* (Figure 4). These results imply that chloroplast ribosomes cannot assemble in this mutant. This conclusion could also be confirmed by the analysis of the levels of chloroplast proteins. Plastid-encoded proteins (such as D1, D2, PetA, PsaA, CF_1_ε, and RbcL) and nucleus-encoded chloroplast proteins (such as PetC, PsaD, and CF_1_γ) which together with their plastid-encode partner proteins form stable complexes are absent or barely detectable in *cdb1* (Figure 2). In contrast, nucleus-encoded chloroplast proteins which do not assemble in complexes with other plastid-encoded proteins accumulate normally in the mutant (Figure 2). We therefore conclude that the absence of *CDB1* compromises chloroplast ribosome assembly, which in turn affects the translation of plastid-encoded mRNAs, notably those of the PEP complex and ultimately impairs chloroplast development during the early stages of seedling growth.

RNA-seq data revealed that almost all chloroplast ribosomal protein genes were upregulated in the *cdb1* mutant except *rps14*, a plastid gene encoding a 30S ribosomal subunit (Supplementary Figure 1). The level of *rps14* transcript in *cdb1* was reduced to ~1/4 of wild type (Supplementary Figure 1). However, this moderate reduction in *rps14* mRNA is unlikely to be the direct reason for the severe deficiency in accumulation of chloroplast ribosomes. Previous studies demonstrated that *RPS14* could also be transcribed to some extent in PEP-deficient mutants (Legen et al., 2002), and reduction of *rps14* transcript can be explained by the absence of the PEP complex. Hence, we propose that CDB1 participates in chloroplast ribosome biogenesis. It may function as a molecular chaperone to assist chloroplast ribosome assembly or maintain the structural stability of ribosomes during their biogenesis. It is also possible that CDB1 is involved in the maturation of the chloroplast rRNAs.

Phylogenetic analysis revealed that the CDB1 paralog CDB1L is present in angiosperms (Figure 6). Knockout of CDB1L results in embryo abortion (Figure 8B). Subcellular localization indicated that CDB1L is dually localized in chloroplasts and mitochondria (Figures 8C–E). The sequence and structural similarity of CDB1L and CDB1 indicates that mitochondria-localized CDB1L may perform a similar function during mitochondrial ribosome biogenesis. Previous reports have confirmed that loss of mitochondrial function usually results in arrested embryo development as observed in mutants deficient in Atp11 and Atp12 that are essential for mitochondrial ATP synthase assembly (Duan et al., 2020) and in AARS proteins that are required for translation in mitochondria (Berg et al., 2005). In *cdb1l* heterozygous plants, some of the seeds can mature but they have a pale color (Figure 8B). Similar seeds were also found in the *cdb1* heterozygous plants (Figure 8B), as well as in the *pmd1*, *pmd2*, *pmd3*, and *pmd4* mutants, in which chloroplast PEP activity is impaired and chloroplast development and biogenesis is arrested (Pyo et al., 2013; Du et al., 2017; Zhang et al., 2017; Wang et al., 2020). These observations suggest that chloroplast development is affected in the pale seeds of *cdb1* and *cdb1l* heterozygous plants. It is possible that chloroplast-localized CDB1L plays an essential role during chloroplast biogenesis and this function cannot be complemented by its CDB1 paralog.

Phylogenetic tree analysis showed that CDB1 homologs can be found in most photosynthetic eukaryotes from green algae to land plants (Figure 6). The conserved residues in the CDB1 family proteins could be clustered to the secondary structure elements of the CDB1 protein structure predicted by AlphaFold2 (Supplementary Figure 2), suggesting that the CDB1 family proteins might have similar biological functions. Interestingly, homologs in angiosperms were further resolved into two clades, containing CDB1 and CDB1L, respectively. The phylogenetic tree showed that CDB1 proteins from lower photosynthetic Viridiplantae are closer to CDB1L than to CDB1 in angiosperms (Figure 6). This suggests that CDB1L proteins originated from green alga and that the CDB1 proteins in monocot and dicot plants probably evolved through gene duplication during the emergence of angiosperms, where they have assumed new functions in chloroplast ribosome biogenesis. The dual-localized CDB1L proteins in angiosperms may have a similar role as CDB1 from lower photosynthetic Viridiplantae, and operate both in chloroplasts and mitochondria.

## Data Availability Statement

The original contributions presented in the study are publicly available. This data can be found here: National Center for Biotechnology Information (NCBI) BioProject database under accession number PRJNA781386.

## Author Contributions

QX conceived the study and designed experiments and produced the figures. WC, JH, and SC performed experiments. WC, JH, SC, LZ, J-DR, LP, and QX analyzed the data. QX, J-DR, and LP wrote the manuscript. QX and LP supervised the whole study. All authors contributed to the article and approved the submitted version.

## Funding

This work was supported by the fund of Shanghai Engineering Research Center of Plant Germplasm Resources (17DZ2252700).

## Conflict of Interest

The authors declare that the research was conducted in the absence of any commercial or financial relationships that could be construed as a potential conflict of interest.

## Publisher’s Note

All claims expressed in this article are solely those of the authors and do not necessarily represent those of their affiliated organizations, or those of the publisher, the editors and the reviewers. Any product that may be evaluated in this article, or claim that may be made by its manufacturer, is not guaranteed or endorsed by the publisher.

## Footnotes

^1^ http://smart.embl-heidelberg.de/

^2^ http://ppdb.tc.cornell.edu/

^3^ http://blast.ncbi.nlm.nih.gov

^4^ https://www.alphafold.ebi.ac.uk/

^5^ http://ppdb.tc.cornell.edu/dbsearch/gene.aspx?id=3387

## Supplementary Material

Supplementary Figure 1 Transcript levels of all sequenced plastid genes. Differential expression of plastid-encoded genes in *cdb1* vs. WT is represented according to the RNA-seq data. The log_2_[fold change(cdb1/WT)] ± SD values are from the three biological replicates.

Supplementary Figure 2 Sequences of CDB1 family proteins from *Arabidopsis thaliana* (CDB1: AT4G37920; CDB1L: AT1G36320), *Populus trichocarpa* (CDB1: XP_024461654.1; CDB1L: XP_024450195.1), *Solanum lycopersicum* (CDB1: XP_004231977.1; CDB1L: XP_004237860.1), *Cinnamomum micranthum f. kanehirae* (CDB1: RWR82510.1; CDB1L: RWR94545.1), *Amborella trichopoda* (CDB1: XP_006856624.2; CDB1L: XP_020521452.1), *Zea mays* (CDB1: NP_001144269.1; CDB1L: NP_001358639.1), *Oryza sativa Japonica* (CDB1: XP_015623683.1; CDB1L: XP_015622979.1), *Physcomitrella patens* (CDB1.1: XP_024388931.1; CDB1.2: XP_024392048.1; and CDB1.3: XP_024372527.1), *Selaginella moellendorffii* (CDB1: XP_002962701.2), and *Chlamydomonas reinhardtii* (CDB1: PNW69832.1). Secondary structure elements above the alignment were generated by ESPript (https://espript.ibcp.fr). Numbers indicate the original amino acid positions in CDB1. Highly conserved residues are represented in white letters with red background.

Supplementary Figure 3 Test of CDB1 and CDB1L antibodies. Mature CDB1 (corresponding to amino acids 63–427 of CDB1) and CDB1L (corresponding to amino acids 13–414 of CDB1L) were used to raise antibodies in rabbits. Recombinant protein of CDB1 and CDB1L (8 ng) was separated by SDS–PAGE and detected with antibodies against CDB1 (**left**) and CDB1L (**right**), respectively. Recombinant proteins with predicted molecular mass are indicated with arrows.
