# Supplementary figures and images for: Stromal Protein Chloroplast Development and Biogenesis1 Is Essential for Chloroplast Development and Biogenesis in Arabidopsis thaliana
Source: Front Plant Sci. 2022 Feb 10;13:815859. doi: 10.3389/fpls.2022.815859 (PMC8866770; doi:10.3389/fpls.2022.815859)

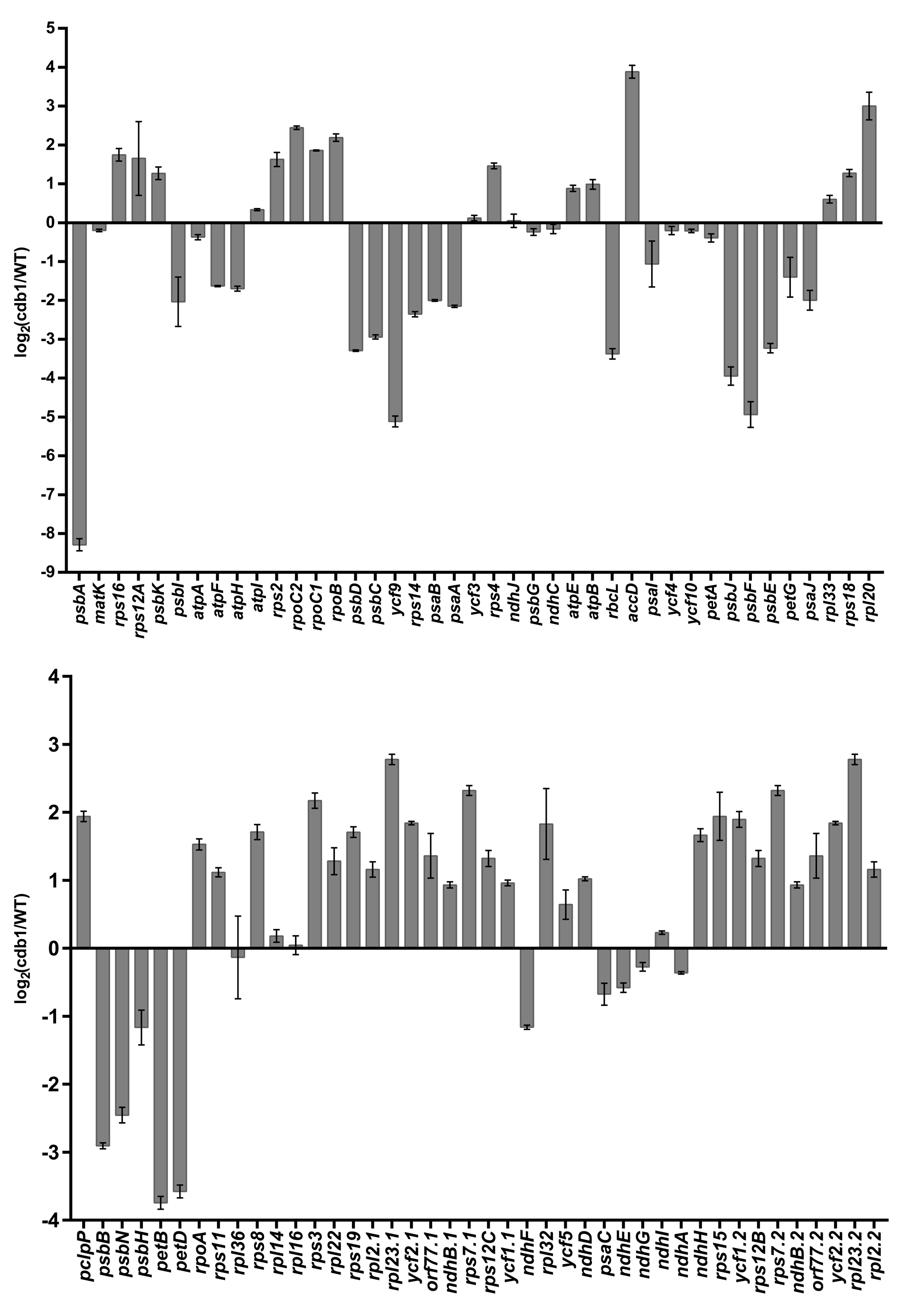

Supplement: Supplementary Figure 1 — Transcript levels of all sequenced plastid genes. Differential expression of plastid-encoded genes in cdb1 vs. WT is represented according to the RNA-seq data. The log2[fold change(cdb1/WT)] ± SD values are from the three biological replicates. [file Image_1.TIF]

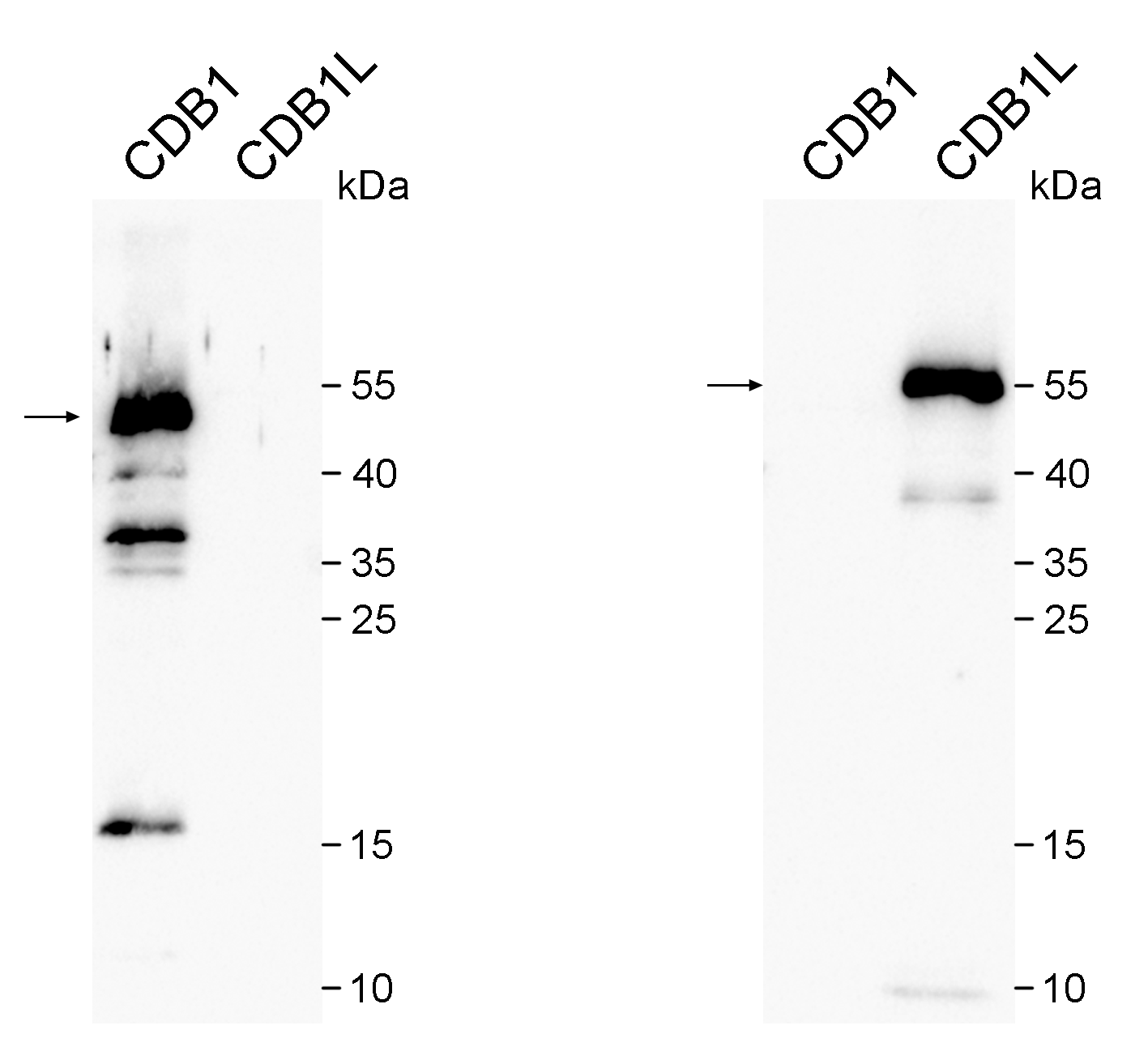

Supplement: Supplementary Figure 3 — Test of CDB1 and CDB1L antibodies. Mature CDB1 (corresponding to amino acids 63–427 of CDB1) and CDB1L (corresponding to amino acids 13–414 of CDB1L) were used to raise antibodies in rabbits. Recombinant protein of CDB1 and CDB1L (8 ng) was separated by SDS–PAGE and detected with antibodies against CDB1 (left) and CDB1L (right), respectively. Recombinant proteins with predicted molecular mass are indicated with arrows. [file Image_3.TIF]
