# Supplementary material for: Stromal Protein Chloroplast Development and Biogenesis1 Is Essential for Chloroplast Development and Biogenesis in Arabidopsis thaliana
Source: Front Plant Sci. 2022 Feb 10;13:815859. doi: 10.3389/fpls.2022.815859 (PMC8866770; doi:10.3389/fpls.2022.815859)
Supplement: Supplementary Figure 2 — Sequences of CDB1 family proteins from Arabidopsis thaliana (CDB1: AT4G37920; CDB1L: AT1G36320), Populus trichocarpa (CDB1: XP_024461654.1; CDB1L: XP_024450195.1), Solanum lycopersicum (CDB1: XP_004231977.1; CDB1L: XP_004237860.1), Cinnamomum micranthum f. kanehirae (CDB1: RWR82510.1; CDB1L: RWR94545.1), Amborella trichopoda (CDB1: XP_006856624.2; CDB1L: XP_020521452.1), Zea mays (CDB1: NP_001144269.1; CDB1L: NP_001358639.1), Oryza sativa Japonica (CDB1: XP_015623683.1; CDB1L: XP_015622979.1), Physcomitrella patens (CDB1.1: XP_024388931.1; CDB1.2: XP_024392048.1; and CDB1.3: XP_024372527.1), Selaginella moellendorffii (CDB1: XP_002962701.2), and Chlamydomonas reinhardtii (CDB1: PNW69832.1). Secondary structure elements above the alignment were generated by ESPript (https://espript.ibcp.fr). Numbers indicate the original amino acid positions in CDB1. Highly conserved residues are represented in white letters with red background. [file Image_2.PDF]

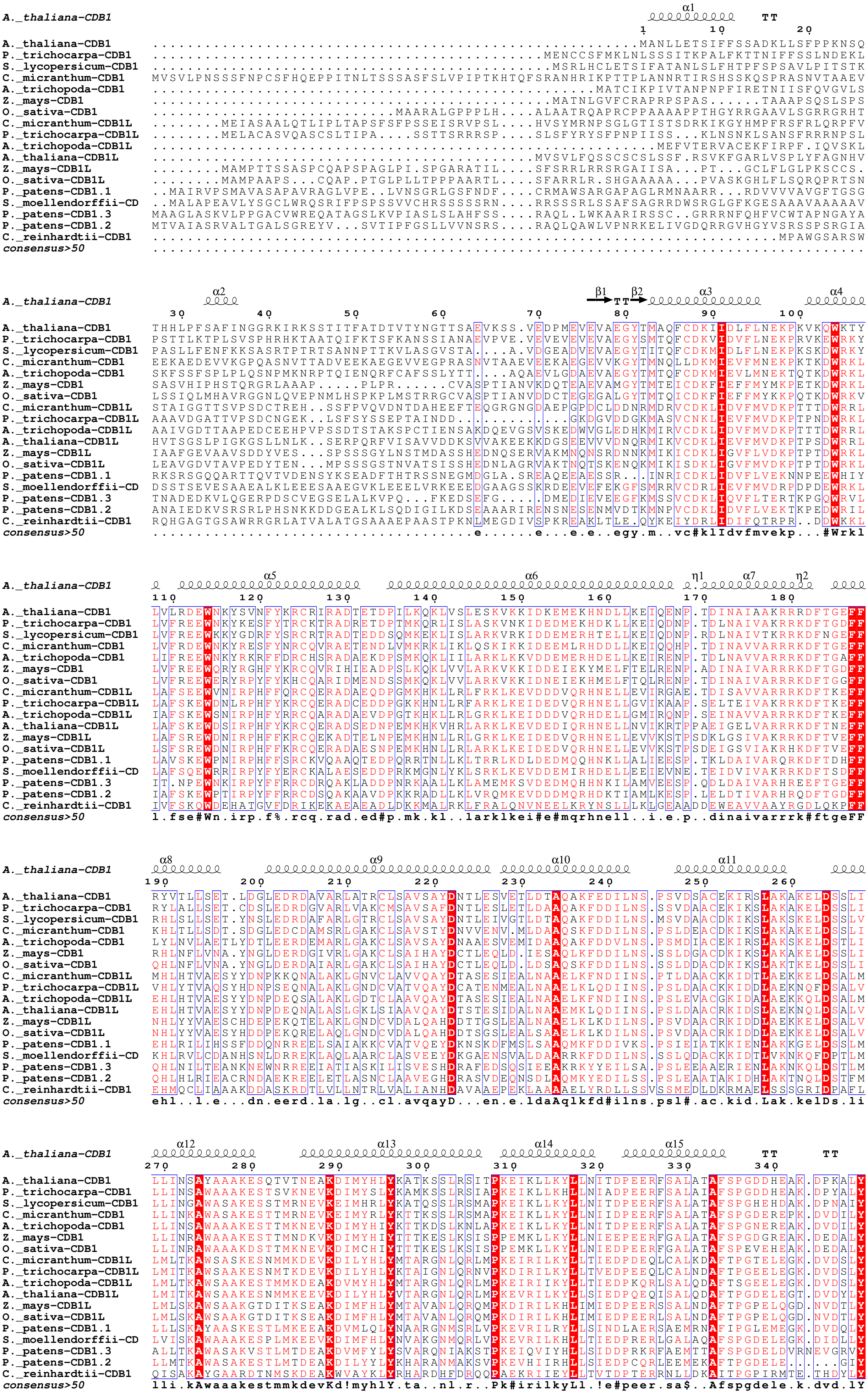
A._thaliana-CDB1 α1 TT
1 10 20
A._thaliana-CDB1 ....MANLLETSIFFSSADKLLSFPPKNSQ
P._trichocarpa-CDB1 ....MENCCSFMKLNLSSSITKPALFKTTNIFFSSLNDEKL
S._lycopersicum-CDB1 ....MSRLHCLETSIFATANLSLFHTPFSPSAVLPITSTK
C._micranthum-CDB1 MVSVLPNSSSFNPCSFHQEPPITNLTSSSASFSLVPIPTKHTQFSRANHRIKPTTPLANNRTIRSHSSKQSPRASNVTAAEV
A._trichopoda-CDB1 ....MATCIKPIVTANPNPFIRETNIISFQVGVLS
Z._mays-CDB1 ....MATNLGVFCRAPRPSPAS......TAAPSQSLSPS
O._sativa-CDB1 ..............................MAARALGPPPLH....ALAATRQAPRCPPAAAAPPTHGYRRGAAVLSGRRGRHT
C._micranthum-CDB1L ........MEIASAALQTLIPLTAPSFSFPSSEISRVPSL....HVSYMRNPSGLETSTSDRKIKGYHMPFRSFRLQRPFV
P._trichocarpa-CDB1L ........MELACASVQASCSLTIPA....SSTTSRRRSP....SLSFYRYSFPNPIISS....KLNSNKLSANSFRRRNPSL
A._trichopoda-CDB1L ....MEFVTERVACEKFIRPF.IQVSKL
A._thaliana-CDB1L ....MVSVLFQSSCSCSLSSF.RSVKFGARLVSPLYFAGNHV
Z._mays-CDB1L ........MAMPTTSSASPCQAPSPAGLPI.SPGARATIL....SFSRRLRRSRGAIISA...PT...GCLFLGLPKSCCS.
O._sativa-CDB1L ........MAMPAAPS...CQAP.PTGLPLLTPPPAARTL....SFARRLR.SHGAAAAA....PVASKGHLFLSQRQPITSN
P._patens-CDB1.1 ..MAIRVPSMAVASAPAVRAGLVPE..LVNSGRLGSFNDF....CRMAWSARGAPAGLRMNAARR...RDVVVVAVGFTGSG
S._moellendorffii-CD ..MALAPEAVLYSGCLWRQSRIFPSPSSVVCHRSSSSSRN....RRSSSRLSSAFSAGRRDWSRGLGFKGESAAAVAAAAV
P._patens-CDB1.3 MAAGLASKVLPPGACVWREQATAGSLKVPIASLAHFSS....RAQL.LWKAARIRSSC..GRRRNFQHFVCWTAPNGAYA
P._patens-CDB1.2 MTVAIASRVALTGALSGREYV...SVTIPFGSLLVVNSRS....RAQLQAWLVPNRKELIVGDQRRGVHGYVSRSSPSRGIA
C._reinhardtii-CDB1 ....MPAWGSARSW
consensus>50 ....

A._thaliana-CDB1 α2 β1 TT β2 α3 α4
30 40 50 60 70 80 90 100
A._thaliana-CDB1 THHLPFSAFINGGRKIRKSSTITFATDTVTYNGTTSAEVKSS.VEDPMEVEVAEGYTMAQFCDKIIDLFLNEKPKVKQWKTY
P._trichocarpa-CDB1 PSTTLKTPLSVSPHRHKTAATQIFKTSFKANSSIANAEVPVE.VEVEVEVEVAEGYSMTQFCDKVIDVFLNEKPRVKEWRKY
S._lycopersicum-CDB1 PASLLFENFKKSASRTPTRTSANNPTTKVLASGVSTA....A.VDGEADVEVAEGYTITQFCDKMIDLFLNEKPKSKDWRKY
C._micranthum-CDB1 EEKAEDEVVKGPQASNVTTADVEEKAEGEVVEGPRASNVTAAEVEEKAEGEVVKGYTMNQLLDKMIEVFMNEKPKTKDWRKL
A._trichopoda-CDB1 SKFSSFSPLPLQSNPMKNRPTQIENQRFCAFSSLYTT...AQAEVLGDAEVAEGYTMTQFCDKMIEVLMNEKTQTKDWRKL
Z._mays-CDB1 SASVHIPHSTQRGRLAAAP.....PLPR......CVASPTIANVKDQTEAEVAMGYTMTEICDKFIEFFMYMKPETKDWRKL
O._sativa-CDB1 LSSIQLMHAVRGGNLQVEPNMLHSPKPLMSTRRGCVASPTIANVDDCTEGEALGYTMTQICDKFIEFFMYKKPQTKDWRKV
C._micranthum-CDB1L STAIGGTTSVPSDCTREH..SSFPVQVDNTDAHEEFTEQGRGNGDAEPGPDCLDDNRMDRVCDKLIDVFMVDKPTTTDWRRL
P._trichocarpa-CDB1L AAAVDGATTVPSDCNGEK..LSFSYSSEPTAINDD.............GKDGVDDGKMASVCNKLIEVFMVDKPTPNDWRKL
A._trichopoda-CDB1L AAIVGDTTAAPEDCEEHPVPSSDTSTAKSPCTIENSAKDQEVGSVSKEDWVGLEDHKMIRVCDKLIDVFMVDKPTTTDWRRL
A._thaliana-CDB1L HVTSGSLPIGKGSLLNLK..SERPQRFVISAVVDDKSVVAKEEKKDGSEEVVVDNQRMIKVCDKLIEVFMVDKPTPSDWRRL
Z._mays-CDB1L IAAFGEVAAVSDDYVES...SPSSSGYLNSTMDASSHEDNQSERVAKMNQNSRDNNKMIKICDKLIGVFLVDKPTPTDWRKL
O._sativa-CDB1L LEAVGDVTAVPEDYTEN...MPSSSGSTNVATSISSHEDNLAGRVAKTNQTSKENQKMIKISDKLIGVFMVDKPTPTDWRKL
P._patens-CDB1.1 RKSRSGQQARTTQVTVDENSYKSEADFTHTRSSNEGMDGLA.SREAQEEAESSR...INRICDKLIDVFLVEKNNPEEWHIY
S._moellendorffii-CD DSSTSEVESAAEALKLEEESAAEGVKLEEELVRKEEEDGAAGSSKRDEEVEFEKGFSMRRVCDRLIEVFMVDRPDPDEWRKL
P._patens-CDB1.3 TNADEDKVLQGERPDSCVEGSELALKVPQ...FKEDSEFG.....DMEDIEVEEGFKMSSVCDKLIQVFLTERTKPGQWRVL
P._patens-CDB1.2 ANAIEDKVSRSRLPHSNKKDDGEALKLSQDIGILKDSEAAARIRENSNESENMVDTKMNPVCDKLIDVFLVEKTKPEDWRIL
C._reinhardtii-CDB1 RQHGAGTGSAWRRGRLATVALATGSAAAEPAASTPKNLMEGDIVSPKREAKLTLE.QYKEIYDRLIDIFQTRPR..DDWKKL
consensus>50 ....e......e...e.e..egy.m..vc#klIdvfmvekp...#Wrkl

A._thaliana-CDB1 α5 α6 η1 α7 η2
110 120 130 140 150 160 170 180
A._thaliana-CDB1 LVLRDEWNKYSVNFYKRCRIRADTETDPILKQKLVSLESKVKKIDKEMEKHNDLLKEIQENP.TDINAIAAKRRRDFTGEFF
P._trichocarpa-CDB1 LVFREEWNKYKESFYTRCKTRADRETDPTMKQRLISLASKVNKIDEDMEKHDKLLKEIQDNP.TDLNAIVAKRRKDFTGDFF
S._lycopersicum-CDB1 LVFREEWKKYGDRFYSRCRTRADTEDDSQMKEKLISLARKVRKIDDEMERHTELLKEIQDNP.RDLNAIVTKRRKDFNGEFF
C._micranthum-CDB1 LVFRDEWNKYRKRFFDRCHSRADAEKDPSMKQKLIKLQSKIKKIDEEMLRHDDLLKEIRENP.TDINAVVARRRKDFTGEFF
A._trichopoda-CDB1 LIFREEWNKYRKRFFDRCHSRADAEKDPSMKQKLIILARKLKKVDDEMERHEDLLKEIRENP.TDLNAVVARRRKDFTGAFF
Z._mays-CDB1 LVFREEWQRYRGHFYKRCQVRIHIEADPSLKQKLVVLARKVKKIDDEIEKYMELFTELRENP.ADINAIVARRRKDFTGDFF
O._sativa-CDB1 LVFREEWERYRPYFYKHCQARIDMENDSSMKQKLVVLARKVKKIDNEIEKHMELFTQLRENP.TDINAIVARRRKDFTGGFF
C._micranthum-CDB1L LAFSEEWVNIRPHFFQRCQERADAEQDPGMKHKLLRLFRKLKEVDDDVQRHNELLEVIRGAE.TDISAVVARRRKDFTKEFF
P._trichocarpa-CDB1L LAFSKEWDNLRPHFYKRCQERADCEDDPGMKHNLLRFARKLKEIDEDVQRHNELLGVIKAAP.SELTEIVAKRRKDFTKEFF
A._trichopoda-CDB1L IAFSKEWSNIRPHFYKRCQARADAEVDPGTKHKLLRLGRKLKEIDDDVQRHDELLGMIRQNP.SEINAVVARRRKDFTKEFF
A._thaliana-CDB1L LAFSKEWDSIRPHFYKRCQERADSEDNPEMKHKVHRLARKLKEVDEDIQRHNELLNVIKRTPPAEIGELVARRRKDFTNEFF
Z._mays-CDB1L LAFSREWDNIRPHFYKRCQEKADTELNPEMKHNLLRLGRKLKEIDEDVQRHNELLEVVKSTPSDKLGSIVAKRRKDFTVEFF
O._sativa-CDB1L LSFSREWDNIRPHFYKRCQERADAESNPEMKHNLLRLARKLKEIDEDVQRHNELLEVVKSTPSDEIGSVIAKRHKDFTVEFF
P._patens-CDB1.1 LAVSKEWPNIRPHFFSRCKVQAAQTEDPQRRTNLLKLTRRLKDLDEDMQQHNKLLALIEESP.TKLDAIVARQRKDFTDHFF
S._moellendorffii-CD LAFSQEWRRIRPYFYERCKALAESEDDPRKMGNLYKLSRKLKEVDDEMIRHDELLEEIEVNE.TEIDVIVAKRRKDFTSDFF
P._patens-CDB1.3 IT.NPEWNKIRPYFFRRCRDRQAKLADDPNRKAALLKLAMEMKSVEDMQHHNKILAMVEESP.QDLDAIVARHREEFTGEFF
P._patens-CDB1.2 IAFSKEWPTIRPYFFRRCRDSQAKAAVDPKRKADLLKLVRQMKEVDDDMQRHDKTIAMLKESP.LELDTIVARHRQDFTGDFF
C._reinhardtii-CDB1 IVFSKQWDEHATGVFDRIKEKAEAEADLDKKMALRKLFRALQNVNEELKRYNSLLLKLGEAADDEWEAVVAAYRGDLQKPFF
consensus>50 l.fse#Wn.irp.f%.rcq.rad.ed#p.mk.kl..larklkei#e#mqrhnell..i.e.p..dinaivarrrk#ftgeFF

A._thaliana-CDB1 α8 α9 α10 α11
190 200 210 220 230 240 250 260
A._thaliana-CDB1 RYVTLLSET.LDGLEDRDAVARLATRCLSAVSAYDNTLESVETLDTAQAKFEDILNS.PSVDSACEKIRSLAKAKELDSSLI
P._trichocarpa-CDB1 RYLALLSET.CDSLEDRDGVARLVAKCMSAVSAFDNTLESLETLDAAQAKFDDILNS.SSVDAACEKIKSLAKAKELDSSLI
S._lycopersicum-CDB1 RHLSLLSET.YNSLEDRDAFARLGTRCLSAVSAYDNTLEIVGTLDTAQAKFDDILNS.MSVDAACDKIKSLAKSKELDSSLV
C._micranthum-CDB1 KHLTLLSDT.SDGLEDCDAMSRLGAKCLSAVSTYDNVVENV.MLDAAQSKFDDILNS.PSLDEACDKIKSLAKAKELDSSLI
A._trichopoda-CDB1 LYLNVLAETLYDTLEERDEMARLGAKCLSAVSAYDNAAESVEMIDAAQAKFDDVLNS.PSMDIACEKIRSLAKSKELDSTLI
Z._mays-CDB1 RHLNFLVNA.YNGLDERDGIVRLGAKCLSAIHAYDCTLEQLD.IESAQLKFDDILNS.SSLDGACDKIKSLAKAKELDSSLI
O._sativa-CDB1 QHLNFLVNA.YNGLDERDAIARLGAKCLSAIHAYDCTLEQLD.LDSAQSKFDDILNS.SSLDDACDKIKSLAKTKELDSSLI
C._micranthum-CDB1L MHLHTVAESYHDNPKKQNALAKLGNVCLAVVQAYDTASESIEALNAAELKFNDIINS.PTLDAACKKIDTLAEKKELDSALM
P._trichocarpa-CDB1L VHLYTVAQSYHDNPSEQNALAKLGNDCVATVQAYDCATENMEALNAAEAELKLQDIINS.PSLDAACKKIDDLAEKNQFDSALV
A._trichopoda-CDB1L EHLHTVAESYYDNPDEQSALAKLGDTCLAAVQAYDTASESIEALNAAELKFNDIINS.PSLEVACGKIDALAEKKQLDSALM
A._thaliana-CDB1L EHLHTVAESYYDNPDEQNALASLGSKLSIAAVQAYDTSTESIDALNAAEMKLQDIINS.PSLDAACRKIDSLAEKNQLDSALV
Z._mays-CDB1L NHLYYVAESCHDEPEKQTELAKLGNDCVDALQAHDDTTGSLEALNAAELKLKDILNS.PSVDAACRKIDDLAEKKELDSALV
O._sativa-CDB1L NHLYYVAESYHDDPEKQRELAQLGNDCVDALQAHDDTSGSLEALSAAELKLKDILNS.PSVDAACRKIDDLAKKKELDSALV
P._patens-CDB1.1 EHLRILIHSSFDDQNRREELSAIAKKCVATVQEYDKNSKDFMSLSAAQMKFDDIVKS.PSIEIATKKIENLAKKGELDSSLM
S._moellendorffii-CD KHLRVLCDANHSNLDRREKLAQLAARCLASVEEYDKGAENSVALDAARRKFDDILNS.SSLQDACKKIDTLVKNKQFDPTLM
P._patens-CDB1.3 QHLNILTEANKNEWNRREEIATIASKILISVESHDRAFEDSQSIEEAKRKYYEILSA.PSLEEAAERIENLAKNKQLDSTLM
P._patens-CDB1.2 QHLHRIEACRNDAEKREELETLASNCLAAVEGHDRASVDEQNSDLAQMKYEDILSS.PSLEEAATAKIDHLAKTNQLDSSLM
C._reinhardtii-CDB1 EHMQCLIAAAKDDASKRDTLVLLNTRLVALIANHDAVAAEPEKLAAAAELYRDLLSSVSSMEDLDKRMAELSSSGRIDPAFL
consensus>50 ehl..l.e...dn.eerd.la.lg..cl.avqayD...en.e.ldaAqlkfd#ilns.psl#.ac.kid.Lak.kelDs.li

A._thaliana-CDB1 α12 α13 α14 α15 TT TT
270 280 290 300 310 320 330 340
A._thaliana-CDB1 LLINSAYAAAKESQTVTNEAKDIMYHLYKATKSSLRSITPKEIKLLKYLLNITDPEERFSALATAFSPGDDHEAK.DPKALY
P._trichocarpa-CDB1 LLINSAWAAAKESTSVKNEVKDIMYSLYKAMKSSLRSIAPKEIKLLKHLLNIADPEERFSALAIAFSPGDDHEAK.DPYALY
S._lycopersicum-CDB1 LLINGAWASAKESSTMRNEVKEIMHRLYKATQSSLRSMAPKEIKLLKYLLNITDPEERFSALATAFSPGHEHDAK.DPNAIY
C._micranthum-CDB1 LLINKAWASAKESTMRNEVKEIMYRLYKTTKSSLRSMAPKEIKLLKYLLNIIDPEERFSALATAFSPGDEREPK.DVDILY
A._trichopoda-CDB1 LLINSAWAAAKESTTMKNEVKDIMYHIYKTTKDSLKNLAPKEIKLLKYLLNITDPEERFSALATAFSPGNEREAK.DVDILY
Z._mays-CDB1 LLINRAWAAAKESTTMNDKVKDIMYHIYTTTKESLKSISPPEMKLLKYLLNIEEPEERFGALATAFSPGDGREAK.DEDALY
O._sativa-CDB1 LLINRAWAAAKDSTTMKNEVKDIMCHIYTTTKESLKSISPPEMKLLKYLLNIEDPEERFGALATAFSPEVEHEAK.DEDALY
C._micranthum-CDB1L LMLTKAWSASKESNMMKDEVKDILYHLYMTARGNLQRLMPKEIRILKYLLTIEDPDEQLCALKDAFTPGTELEGK.DVDSLY
P._trichocarpa-CDB1L LMITKAWSAAKESNMTKDEVKDILYHLYKTAIGNLQRNVPKDIRILKYLLTVEDPEEQLCALNDAFTPGEELEGK.DVDSLY
A._trichopoda-CDB1L LMLTKAWSAAKESTMMKDEAKDVMYHLYMTARGNLQRLMPKEIRIIKYLLTIEDPKQRLSALQDAFTSGEELEGK.DVDSLY
A._thaliana-CDB1L LMITKAWSAAKESNMMKEEVKDILYHLYVTARGNLQRLMPKEVRILKYLLSIEDPQEQISALQDAFTPGDELEGT.DVDYLY
Z._mays-CDB1L LMLSKAWSAAKGTDITKSEAKDIMFHLYMTAVANLQRQMPKDIRILKHLLIMIEDPEERSSALNDAFTPGPELQGD.NVDTLY
O._sativa-CDB1L LMLSKAWSAAKGTDITKSEAKDIMFHLYMTAVANLQRQMPKDIRILKHLLIMIEDPEERLSALNDAFTPGPELQGD.NVDTLY
P._patens-CDB1.1 LLLSKAYASAKESTLMKEEAKDVMLQLYNAARGNMSRLVPKEVRILRYLLSINDLAERSAEMRNAFIPGLEMEGK.DVDQLY
S._moellendorffii-CD LVISKAWAAAKESPLMKEEVKDVMFHLYNVAKGNMQRLVPKEVRILKHLLTIEDPRERLGALAQAFSPGDELEGK.DIDLLY
P._patens-CDB1.3 ALLTKAWASAKVSTLMTDEAKDVMFYLYSVARQNIAKSTPKEIQVIYHLLSIDDPRKRFAEMTTAFSPGDELDVRNEVGRVY
P._patens-CDB1.2 LLMTKAWASAKESTLMTDEAKDILYYLYKHARANMAKSVPKEVHIIRHLLSIEDPCQRLEEMEKAFTPGDELE.....GIY
C._reinhardtii-CDB1 QISAKAYGAARDTNMSKDEAKWVAYKLYRHARDHFDRQQPAEKRIIEYLLTVTDPTERRNLLDKAITPGPIRMTE.THDYLY
consensus>50 lli.kAwaaakestmmkdevKd!myhlY.ta..nl.r..Pk#irilkyLl.!e#peer.sa$..Afspgdele.k.dvd.lY

```
                            α16                      α17            α18              η3
A._thaliana-CDB1
                    350         360        370        380        390        400        410        420
A._thaliana-CDB1        TTPKELHKWIKIMLDAYHLNKEETD..IKEAKQMSQPIVIQRLFILKDTIEDEYLDKKTIVADET.PKKEEEDTTIEDFLN
P._trichocarpa-CDB1     TTPKELHKWIKIMLDAYHLNKEDTD..IKEAKQMSQPVIIQRLFILKETIEEEYLEKTTFQTRPE.GDTKSED........
S._lycopersicum-CDB1    TSPKELHKWIKIMLDAYNMNKEETE..IREAKQLDQPMVIQRLSILKETVEAEYLEKE...ANTE.KDSQPEETVI.....
C._micranthum-CDB1      TTPKELHKWIKIMLDSYQLHKEETD..LREAREMSHPMVIQRLFILKETIETEYMRQPNETADPEEKDPEAKETEEDDFTI
A._trichopoda-CDB1      TTPKELHKWITIMLDAYHINKEETD..LREARQMTQPVVIQRLFILKETIEEEYMREKTEAK....KDPEPEE........
Z._mays-CDB1            TTPSELHKWIKMLLDSYHLNKEETD..FMDARKMSDPVIIQRLTLLKEMIEEEYMKRYIHPEEEEEPMDDS.........
O._sativa-CDB1          TTPNELHKWTKMMLDSYHLNKEETD..FMDARKMSDPVIIQRLTLLKETIEEEYMKKYIHPEEQESEDDEDSEE.......
C._micranthum-CDB1L     TTPEKLFTWIRTVVDAYHFSREGTL..IREARDLMNPKIIQKLEELKKLVQDNFM..........................
P._trichocarpa-CDB1L    TTPEKLHTWIKAMVDAYHLSQDGTL..MREARDLMNPKIIKKMEELKKLVEDHFM..........................
A._trichopoda-CDB1L     TTPERLFTWVQTVLDAYRFSREGTL..IREARDLMSPKIIQKLEELKKVLQNNFM..........................
A._thaliana-CDB1L       TTPEHLQSLMKTVLEAYHFSREGSL..VKEAKDLMHPELIAKIEQLKKLVEKKYM..........................
Z._mays-CDB1L           TSPEALHTWARAIVDAYYNSREGTL..IGQARDMMNPKIIKRVEEIVKIIKDKYL..........................
O._sativa-CDB1L         TSPEVLHTWASAIVDAYYNSREGTL..LRQARDLMNPKIIKRIEEILKLIKDKYL..........................
P._patens-CDB1.1        TTPEALLMTIQNLLDAVDTNRKGAL..LKEARKLIQPSVLGRLRAIKQELESQYM..........................
S._moellendorffii-CD    TTPNRLYRQVLFILDAYYSNKEKTL..IQEAQKLMDPLVISRLEILRDVIKTEFL..........................
P._patens-CDB1.3        TSPDSLLKWINIILDAFYLNKRGTL..IKEAQSLLNPTVISRLEVLRDVIKEQFIDHDMSFENP.................
P._patens-CDB1.2        TEPEKLLEWISAVLEAFYSKQKGTL..IKEAEGMLNPSFISRLVTLRDLIVKYFI..........................
C._reinhardtii-CDB1     STPQRLFLVLDNTLRAYHLMRDGAAKNLNTPEAAVLPRKVRVMMELRDDIIRRYL..........................
consensus>50            ttPe.Lh.wi..mldayhlnkegtl..i.eardlmnP.iiqrl..lke.iede%m..........................
```
