# Supplementary material for: Stromal Protein Chloroplast Development and Biogenesis1 Is Essential for Chloroplast Development and Biogenesis in Arabidopsis thaliana
Source: Front Plant Sci. 2022 Feb 10;13:815859. doi: 10.3389/fpls.2022.815859 (PMC8866770; doi:10.3389/fpls.2022.815859)
Supplement: Supplementary file 4 [file Data_Sheet_1.docx]

**Supplementary Table 1. TMM values of plastid genes in Figure 3C.**

|  | Symbol | WT TMM | cdb1 TMM | log_2_FoldChange | pvalue |
| --- | --- | --- | --- | --- | --- |
| Class I | *psbA* | 605813.511 | 1914.664 | -8.365653758 | 0 |
| Class I | *psbH* | 565.206333 | 247.7556667 | -1.240617671 | 2.36E-15 |
| Class I | *petB* | 17958.3547 | 1344.237667 | -3.793173753 | 2.38E-239 |
| Class I | *petD* | 8799.84567 | 740.073 | -3.625807035 | 9.71E-263 |
| Class I | *psaB* | 26878.224 | 6713.924667 | -2.05780384 | 1.57E-93 |
| Class I | *ndhA* | 1237.81933 | 963.6593333 | -0.416490156 | 3.39E-06 |
| Class I | *rbcL* | 109435.686 | 10471.45633 | -3.444420105 | 2.13E-137 |
| Class II | *atpI* | 1698.56633 | 2140.638333 | 0.28035549 | 0.0010969 |
| Class II | *rps16* | 19.6036667 | 77.27566667 | 1.922241454 | 7.63E-11 |
| Class II | *ndhF* | 232.95 | 104.1233333 | -1.217049678 | 2.00E-38 |
| Class II | *clpP* | 1282.428 | 4908.835667 | 1.884119745 | 5.41E-82 |
| Class II | *ycf1* | 669.724 | 1302.291667 | 0.906127548 | 1.24E-23 |
| Class III | *accD* | 74.5763333 | 1085.471 | 3.811982418 | 1.59E-170 |
| Class III | *rpoA* | 192.434333 | 552.762 | 1.470278895 | 9.42E-32 |
| Class III | *rpoB* | 178.503 | 812.482 | 2.13364236 | 2.99E-120 |
| Class III | *rpoC1* | 812.457667 | 2944.642667 | 1.803039634 | 3.26E-89 |
| Class III | *rpoC2* | 238.617667 | 1295.25 | 2.386971941 | 3.63E-174 |
| Class III | *ycf2* | 239.861 | 860.458 | 1.789898208 | 1.30E-82 |

**Supplementary Table 2. Segregation ratio in the progeny of the *CDB1L*/*cdb1l* mutant****.**

| Materials | Plants tested | Het : WT observed | Het : WT ratio tested | χ^2^ | P |
| --- | --- | --- | --- | --- | --- |
| F_2_ | 96 | 63 : 33 | 64 : 32 | 0.047 | 0.83 |

Het: heterozygous plants; WT: wild type plants

**Supplementary Table 3. Primers used in this study.**

| Primer name | Primer sequence 5' to 3' | Comment |
| --- | --- | --- |
| CDB1-TF | TCCTTCTTTGGAGTTTCATCAGC | Genotyping primer |
| CDB1-TR | CAAGAGACGCAGAGACTTTACGG |  |
| CDB1L-TF | CTTGATGCTGCCTGTAGAAAGATTG |  |
| CDB1L-TR | AGTGTAACTGAACCAGACCGAAAAG |  |
| CDB1-Ab-F | ACT**GAATTC**GCGGAAGTAAAAAGCTCTGTTG | Primers for antibody production |
| CDB1-Ab-R | CAA**CTCGAG**GTTCAAAAAATCTTCAATAGTCG |  |
| CDB1L-Ab-F | TGT**GAATTC**TCACTTTCTAGCTTTCGCTCTG |  |
| CDB1L-Ab-R | TTT**CTCGAG**CATATACTTCTTTTCAACAAGC |  |
| CDB1-GFP-F | GGA**ACTAGT**ATGGCGAATTTACTGGAAACATC | Primers for subcellular localization |
| CDB1-GFP-R | CAC**CCGCGG**CAGTTCAAAAAATCTTCAATAG |  |
| CDB1L-GFP-F | GCC**TCTAGA**ATGGTTTCAGTGTTATTCCAATC |  |
| CDB1L-GFP-R | GTT**CCGCGG**CACATATACTTCTTTTCAACAAG |  |
| probe_rrn16_for | AGTCATCATGCCCCTTATGC | Primers for RNA hybridization probes |
| probe_rrn16_rev | CAGTCACTAGCCCTGCCTTC |  |
| probe_rrn23_for | GTTCGAGTACCAGGCGCTAC |  |
| probe_rrn23_rev | CGGAGACCTGTGTTTTTGGT |  |
| probe_rrn4.5_for | GAAGGTCACGGCGAGACGAGCC |  |
| probe_rrn4.5_rev | GTTCAAGTCTACCGGTCTGTTAGG |  |
| probe_rrn5_for | TATTCTGGTGTCCTAGGCGTAG |  |
| probe_rrn5_rev | ATCCTGGCGTCGAGCTATTTTTCC |  |
| CDB1-1301-F | GAA**GGTACC**GAAACAGAGACACTCAAAGAAGAGC | Primers for complementation tests |
| CDB1-1301-R-COM | CCA**GTCGAC**TCAGTTCAAAAAATCTTCAATAGTC |  |
| CDB1-1301-R-HA | CAA**GTCGAC**GTTCAAAAAATCTTCAATAGTCG |  |
| CDB1L-1301-F | TGT**GGTACC**ATACTGTAATCTTTAACAGGTTGGC |  |
| CDB1L-1301-R-COM | AGT**GTCGAC**TCACATATACTTCTTTTCAACAAGC |  |
